# Amniotic fluid mesenchymal stem cells repair mouse corneal cold injury by promoting mRNA N4-acetylcytidine modification and ETV4/JUN/CCND2 signal axis activation

**DOI:** 10.1007/s13577-020-00442-7

**Published:** 2020-10-03

**Authors:** Xinfeng Fei, Yuying Cai, Feng Lin, Yongyi Huang, Te Liu, Yan Liu

**Affiliations:** 1 Department of Ophthalmology, Shanghai Fourth People’s Hospital Affiliated to Tongji University School of Medicine, 100 Haining Road, Shanghai, 200080 China; 2 Department of Ophthalmology, Shanghai General Hospital, School of Medicine, Shanghai JiaoTong University, Shanghai, 200080 China; 3 National Clinical Research Center for Eye Diseases, Shanghai General Hospital, Shanghai, 200080 China; 4 Shanghai Key Laboratory of Ocular Fundus Diseases, Shanghai General Hospital, Shanghai, 200080 China; 5 Shanghai Engineering Center for Visual Science and Photomedicine, Shanghai General Hospital, Shanghai, 200080 China; 6 Shanghai Engineering Center for Precise Diagnosis and Treatment of Eye Diseases, Shanghai General Hospital, Shanghai, 200080 China; 7 School of Environmental and Chemical Engineering, Shanghai University, Shanghai, 200444 China; 8 Shanghai Geriatric Institute of Chinese Medicine, Shanghai University of Traditional Chinese Medicine, Building C, 365 Xiangyang Road, Shanghai, 200031 China

**Keywords:** Corneal cold injury stem cell repair, mRNA N4-acetylcytidine modification, NAT, Etv4/Jun/Ccnd2 pathway

## Abstract

Severe corneal injury is one of the main causes of loss of visual function. Mesenchymal stem cells (MSCs) have the ability to repair damaged cells in vivo. The present study aimed to explore whether MSCs could function as a cell therapy tool to replace traditional methods to treat corneal injury. CD44 + /CD105 + mesenchymal stem cells isolated from mouse amniotic fluid (mAF-MSCs) were injected into mice after cryoinjury to induce corneal endothelial cell injury. Histopathological assays indicated that mAF-MSCs could promote the growth of corneal epithelial cells, reduce keratitis, and repair the corneal damage caused by low temperature. cDNA microarray analysis revealed that the mAF-MSCs affected the expression patterns of mRNAs related to cell proliferation and differentiation pathways in the mice after transplantation. The results of quantitative real-time PCR and western blotting revealed that NAT12, NAT10, and the ETV4/JUN/CCND2 signaling axis were elevated significantly in the mAF-MSC-transplantation group, compared with those in the phosphate-buffered saline-treated groups. High performance liquid chromatography–mass spectroscopy results revealed that mAF-MSCs could promote mRNA N4-acetylcytidine (ac4C) modification and high expression of *N*-acetyltransferase in the eyeballs. RNA immunoprecipitation-PCR results showed that a specific product comprising *Vegfa*, *Klf4*, *Ccnd2*, *Jun*, and *Etv4* mRNA specific coding region sites could be amplified using PCR from complexes formed in mAF-MSC-transplanted samples cross-linked with anti-ac4C antibodies. Thus, mouse amniotic fluid MSCs could repair the mouse corneal cold injury by promoting the ETV4/JUN/CCND2 signal axis activation and improving its stability by stimulating N4-acetylcytidine modification of their mRNAs.

**Electronic supplementary material:**

The online version of this article (10.1007/s13577-020-00442-7) contains supplementary material, which is available to authorized users.

## Introduction

The cornea is divided into five layers, which are listed from posterior to anterior as follows: Epithelium, lamina elastica anterior (Bowman membrane), stroma, lamina elastica posterior (Descemet membrane), and endothelium [1–6]. The endothelium is arranged as a single layer of hexagonal smooth corneal endothelial cells (CECs). CECs line the posterior surface of the cornea and face the anterior chamber. They also regulate the passage of fluids and solutes across the posterior surface of the cornea to maintain a slightly dehydrated state, which is required for transparency and refraction [1–6]. CECs are critical to maintain the structure and function of the cornea; they also possess a barrier function, regulate osmotic pressure, and control the active transport of fluid out of the cornea via the activity of the Na + /K + -ATPase to maintain normal corneal metabolism [1–5]. CECs do not regenerate, because they are end-stage differentiated cells [1–5]. Damage to a large number of CECs causes edema, corneal degeneration, corneal decompensation, and possibly, total blindness [1–5]. Corneal damage might be caused by a number of factors, including mechanical injury, exposure to solar radiation over an extended period of time, exposure to chemicals, and freezing [7, 8]. Corneal injury caused by freezing of CECs is common in the cold areas of the world and can damage vision [6]. Severe corneal injury leads to permanent corneal swelling and bullous keratopathy, and is considered one of the main causes of loss of visual function. Although corneal transplantation is a good method to treat corneal injury, the application and development of this treatment method is limited for various reasons, especially a lack of corneal donors [6]. Therefore, it is difficult to repair and treat corneal injury caused by freezing. One of the main purposes of this study is to find other biomaterials besides corneal transplantation, which should be easy to obtain, safe and efficient, and can significantly repair corneal freezing injury. At the same time, the molecular biological mechanism of above biomaterials (such as stem cells, etc.) differentiating into corneal epithelial like cells and further realizing corneal repair after transplantation is revealed. Our research results will provide a new idea for the repair of frozen corneal injury and the development of cell preparations.

Mesenchymal stem cells (MSCs) originate during early mesoderm and ectoderm development, and exist in a variety of tissues, such as the bone marrow, umbilical cord blood and umbilical cord, placenta, and adipose tissue. They have varying somatic differentiation potentials and unique cytokine secretion functions that are characteristics of pluripotent stem cells [9–12]. Stem cell surface markers, including cluster of differentiation (CD)29, CD44, CD49f, CD90, CD105, and CD117, are highly expressed in MSCs [9–12]. Extensive research has indicated that MSCs possess strong pluripotent characteristics. For example, under specific conditions, MSCs can be induced to differentiate into a variety of somatic cell types, including adipocytes, osteoblasts, chondrocytes, neurons, endotheliocytes, pulmonary epithelial cells, hepatocytes, islet cells, cardiac myocytes, and insulin-producing cells [9–12].

Posttranscriptional modifications of RNA represent an emerging class of regulatory elements in human biology [13, 14]. N4-acetylcytidine (ac4C) is a highly conserved RNA modification and was the first acetylation event described in mRNA. Ac4C-modified mRNA has been demonstrated to be involved in the regulation of mRNA stability, processing, and translation [13, 15]. Modification of tRNA anticodons plays a critical role in ensuring accurate translation. The ac4C modification is present at the anticodon first position (position 34) of bacterial elongator tRNA^Met^ [16–18]. In addition, *Bacillus subtilis ylbM* (tmcAL) is as a novel gene responsible for ac4C34 formation [16–18]. In a variety of human cells, recent studies have detected ac4C in poly(A) RNA at a similar level to 5′ 7-methylguanosine (m7G) cap, which suggests that ac4C is abundant in mRNA and has important regulatory functions [14, 15, 19–21]. Arango et al. also demonstrated that repeating CXX motifs (several obligate cytidines separated by two non-obligate nucleotides) are highly enriched in ac4C peaks [22]. Mammalian *N*-acetyltransferase 10 (NAT10) is a lysine acetyltransferase that targets microtubules and histones, and plays an important role in cell division [14]. NAT10 is highly expressed in malignant tumors, and is a promising target for therapies against laminopathies and premature aging [14]. Ito et al. reported that NAT10 is an ATP-dependent RNA acetyltransferase responsible for formation of ac4C at position 1842 in the terminal helix of mammalian 18S Ribosomal RNA [14]. Arango et al. also indicated that the *NAT10* gene could improve mRNA stability and translation by maintaining acetylation to promote target gene expression [22].

In our previous study, we indicated that cryoinjury, or injury caused by extremely low temperatures, leads to damage and apoptosis of mouse corneal endothelial cells by activation of the serine/threonine kinase 11 (STK11)-P53 signaling pathway, phosphorylation of P53 serine 15, and *p21* gene transcription [6]. Therefore, in the present study, on the one hand, we aimed to explore whether CD44^+^/CD105^+^ MSCs isolated from mouse amniotic fluid (mAF-MSCs) were effective to treat a freezing-induced mouse model of corneal endothelial cells injury (CECI). On the other hand, we aimed to clarify its in-depth treatment mechanism.

## Materials and methods

### Isolation and enrichment of CD44^+^/CD105^+^ mAF-MSCs

Samples of mAFs were obtained from 10 pregnant mice on the 18^th^ day of gestation. The CD44^+^/CD105^+^ subpopulation of cells was extracted from the mAFs using 4 μl of primary monoclonal antibodies (rabbit anti-mouse CD44-fluorescein isothiocyanate (FITC) and rabbit anti-mouse CD105-phycoerythrin (PE); eBioscience Inc, San Diego, CA, USA) and stored at 4 °C in phosphate-buffered saline (PBS) for 30 min in a volume of 1 ml, as previously described [23]. Subsequently, the cells were washed twice in PBS, incubated with secondary monoclonal antibodies (goat anti-rabbit coupled to magnetic microbeads; Miltenyi Biotec, Auburn, CA, USA) at 10 °C in PBS for 15 min, and then washed twice with PBS. The CD44^+^/CD105^+^ subpopulation of cells were plated at 1 × 10^6^ cells/ml in Dulbecco’s modified Eagle’s medium (DMEM):F12 (1:1) (Gibco, Gaithersburg, MD, USA), supplemented with 10 ng/ml basic fibroblast growth factor (bFGF), 10 ng/ml epidermal growth factor (EGF) (all from Sigma-Aldrich, St Louis, MO, USA), 10% fetal bovine serum, and 2 mM l-glutamine (all from Gibco). In this experiment, all CD44^+^/CD105^+^ cells were cultured under the same conditions in a humidified incubator, at 37 °C and 5% CO_2_, until the cells were 80% confluent. Cells were not used for further experiments after the third passage. All the animal experiments were conducted in accordance with the guidelines of the NIH for the care and use of laboratory animals. The study protocol was also approved by the Committee on the Use of Live Animals in Teaching and Research, Shanghai Geriatric Institute of Chinese Medicine, Shanghai, China.

### RNA extraction and analysis by quantitative real-time reverse transcription PCR (qRT-PCR)

Briefly, total cellular RNA was extracted using the Trizol Reagent (Invitrogen, Waltham, MA, USA) according to the manufacturer’s protocol. The RNA was then reverse-transcribed into cDNA using a ReverTra Ace-α First Strand cDNA Synthesis Kit (TOYOBO, Osaka, Japan). cDNA was then amplified using quantitative real-time PCR (qPCR). QPCR was conducted using a RealPlex4 real-time PCR detection system from Eppendorf Co. Ltd (Hamburg, Germany), with the SyBR Green RealTime PCR Master Mix and detection dye (TOYOBO). QPCR amplification was performed over 40 cycles. The qPCR cycle consisted of denaturation at 95 °C for 15 s and annealing at 58 °C for 45 s. The target cDNA was evaluated by relative quantification. A comparative cycle threshold (Ct) method was used to determine the gene expression relative to a control (calibrator, 18S rRNA) and steady-state mRNA levels were reported as an n-fold difference relative to that of the calibrator. For each sample, the marker gene Ct values were normalized using the formula ΔCt = Ct_genes—Ct_18S rRNA. To determine the relative expression levels, the following formula was used: ΔΔCt = ΔCt_treated_group—ΔCt_control_group. Three biological replications were performed for each reaction. The 2^−ΔΔCt^ method was applied to measure the relative marker expression. The internal control, 18S rRNA, served as a normalizer. Data were analyzed on triplicate samples by the GraphPad Prism Version 5.00 (GraphPad Software, Inc.), and presented as relative expression of each gene, compared with PBS-transplant group. All data are expressed as mean ± standard error (*X* ± SE). Statistical analysis was performed using paired or unpaired *t* tests as appropriate. A *P* value of < 0.05 was considered significant. The sequences of the real-time PCR primers are shown in supplementary Table S1.

### Flow cytometry (FCM) analysis

All the CD44^+^/CD105^+^ subpopulation of cells were collected and washed with PBS by centrifugation. The cells were suspended at a density of 1 × 10^4^ cells/ml. The cell suspension was incubated with a primary antibody recognizing a cell surface antigen or isotype control antibody (CD29, CD90, or CD105 conjugated with FITC; eBioscience) on ice in Dulbecco’s PBS (DPBS) containing 10% bovine serum albumin (BSA). The sensitivity of the flow cytometer was adjusted using the cells treated with an isotype control antibody (mouse IgG1-FITC, eBioscience) to rectify non-specific binding. Antibody staining by FCM was analyzed on a FACS Aria system (Quanta SC, Beckman Coulter INC., Indianapolis, IN, USA).

### Animals, cryoinjury treatment, and in vivo transplantation experiments

Female C57BL/6 mice (*n* = 20), between 4 and 5 weeks of age, were initially bred and maintained under standardized conditions in the Animal Research Centre at Shanghai General People’s Hospital of Shanghai JiaoTong University, China. All experiments were authorized (Permit SJTAEC201401) by the Animal Ethics Committee of Shanghai JiaoTong University and conducted in compliance with the Experimental Animal Regulations of the National Science and Technology Commission, China. Mice weighing 22–35 g were randomly divided into four groups. All mice were housed for 14 days under standard light–dark cycle with food and water provided ad libitum. The animal room was maintained at 23 °C and 55% relative humidity. Cryoinjury was induced as previously described [8]. A cryoprobe with a diameter of 2.5 mm was frozen in liquid nitrogen. It should be noted that the cryoprobe had a diameter similar to that of the C57BL/6 mouse cornea (2.6 mm). The cryoprobe was then placed on the mouse cornea three times at 1-min intervals. The mice were then randomly divided into two groups: A negative control group (*n* = 10) injected with PBS in the corneal endothelium, and an experimental group (*n* = 10 mice) injected with CD44^+^/CD105^+^ mAF-MSCs in the corneal endothelium. One week after corneal injury, every mouse in the experimental group was injected with 10 μl of either cell suspension (approximately 1 × 10^3^ cell spheres/μl) or PBS. Additional experiments were performed on the animals in both groups at 10 days after transplantation.

### Western blotting analysis

Briefly, cells were removed from culture flasks using cell scrapers and then lysed in pre-cooled (4 °C) cell lysis buffer (Beyotime Institute of Biotechnology, Haimen, China). Subsequently, the sample was centrifuged at 436 × *g* for 5 min. The supernatant was placed in a fresh 1.5-ml reaction tube and used for further analysis. The protein concentration (50 μg/μl) was determined by the bicinchoninic acid assay. The total protein extract (15 μl) was resolved by 12% sodium dodecyl sulfate (SDS)-polyacrylamide gel electrophoresis, and transferred onto polyvinylidene difluoride membranes (EMD Millipore, Billerica, MA, USA). The membranes were blocked with a Tris-buffered solution containing 10% calf serum (Beyotime Institute of Biotechnology, Haimen, China). After washing four times with Tris-buffered saline containing Tween-20 (TBST; Sigma-Aldrich) at room temperature, the membranes were incubated with primary antibodies (Supplementary Table S2) overnight at 4 °C. Following sufficient washes, the membranes were incubated with appropriate secondary antibodies (Supplementary Table S2) for 1 h. After additional washes (15 min each) with TBST at room temperature, the immunoreactive proteins were detected using enhanced chemiluminescence with the Bio-Rad Gel Imaging system (ECL kit; Pierce Biotechnology, Inc., Rockford, IL, USA) and exposure to Kodak Biomax XAR-5 films (Sigma-Aldrich).

### Hematoxylin–eosin (HE) staining

Tissue samples were fixed with 4% paraformaldehyde, dehydrated, and embedded in paraffin. Slices with a thickness of 4 μm were cut using a paraffin slicer and fixed to glass slides. Xylene was used for dewaxing, and ethanol gradient dehydration was performed. Hematoxylin solution was used to stain the cells at room temperature for 5 min, 1% hydrochloric acid ethanol was used for differentiation for 30 s, light ammonia water was added to return to blue for 1 min, and distilled water was used to rinse for 5 min. Subsequently, eosin solution was applied to the sections at room temperature for 2 min, and rinsed with distilled water for 2 min. Ethanol gradient decolorization was performed. Xylene was allowed to permeate for 2 min. Finally, the slide was sealed using neutral gum.

### Immunofluorescence stain assay

Briefly, all fresh tissues were soaked at room temperature and fixed in 4% paraformaldehyde (Sigma-Aldrich) for 30 min. Ethanol gradient dehydration, paraffin embedding, slicing (thickness 6 μm), and dewaxing using xylene were performed. The tissue sections were sealed at 37 °C for 30 min using immunohistochemical blocking solution (Beyotime Biotechnology Co., Ltd., Zhejiang, China). The blocking solution was discarded, and immunohistochemical cleaning solution (Beyotime Biotechnology Co., Ltd) was added to rinse the tissue sections at room temperature three times for 5 min each. The first antibody (supplementary Table S2) was added and incubated at 37 °C for 45 min. The antibody was discarded, and immunohistochemical cleaning solution was added to rinse the tissue at room temperature three times for 5 min each. The secondary antibody (Supplementary Table S2) was added and incubated at 37 °C for 45 min. The antibody was discarded before adding immunohistochemical washing solution to rinse the tissue at room temperature three times for 5 min each. Finally, immunofluorescence sealing solution (Sigma-Aldrich) was added to seal the slides.

### cDNA microarray analysis

The RNA samples from CECI mice eyeballs transplanted with mAF-MSCs or PBS were pooled and used for microarray analysis. Samples were labeled using the Agilent Low RNA Input Fluorescent Linear Amplification kit, according to the manufacturer’s instructions (Agilent Technologies, Santa Clara, CA, USA). Either Cy3-dCTP or Cy5-dCTP was added to 5 μg of total RNA during reverse transcription. Fluorescently labeled cDNA probes were mixed in 30 μl of hybridization buffer (3 × SSC, 0.2% SDS, 5 × Denhardt's solution, and 25% formamide) and then applied to the microarray (Genenergy mouse mRNA microarray V2.0, Genenergy, Shanghai, China). Subsequently, slides were incubated at 42 °C for 16 h. After hybridization, the slides were washed once in 0.2% SDS/2 × SSC at 42 °C for 5 min, followed by washes in 0.2 × SSC at room temperature for 5 min. Finally, the fluorescence images of the hybridized microarray were scanned using an Agilent Whole Mouse Genome 4 × 44 microarray scanner system. The images and quantitative data of the gene-expression levels were analyzed using Agilent's Feature Extraction (FE) software, version 9.5.

### Dot blotting

The total RNAs from each group were spotted on a Hybond-N + membrane. The spotted RNAs were then cross-linked to the membrane using a UV Crosslinker, the membrane was blocked in 5% BSA, and subsequently incubated with rabbit anti-N4-acetylcytidine (ac4C) antibodies [EPRNCI-184-128] (Abcam, Cambridge, MA, USA; ab252215) and HRP-conjugated anti-rabbit secondary antibody (CST, Danvers, MA, USA), and finally developed using enhanced chemiluminescence reagents and exposed to imaging film.

### RNA immunoprecipitation (RIP)-PCR

RIP experiments were performed using the Magna RIP RNA-Binding Protein Immunoprecipitation Kit (Millipore, Bedford, MA, USA). Briefly, cells from all groups were lysed (500 μL per plate) in a modified cell lysis buffer used for western blotting and IP (20 mM Tris, pH 7.5, 150 mM NaCl, 1% Triton X-100, 1 mM EDTA, sodium pyrophosphate, β-glycerophosphate, Na_3_VO_4_, and leupeptin) (Beyotime institute of Biotechnology). After lysis, each sample was centrifuged to clear the insoluble debris and then pre-incubated with 20 μg protein A agarose beads (Beyotime institute of Biotechnology) by rocking for 30 min at 4 °C, followed by centrifugation and transfer to a fresh 1.5 mL tube. The rabbit anti-ac4C antibodies [EPRNCI-184–128] (1:100, Abcam, ab252215) or rabbit anti-NAT10 antibodies [EPR18663] (1:100, Abcam, ab194297) were added and incubated for 90 min before the re-addition of 20 μg of protein A agarose beads to capture the immune complexes. The agarose beads were then washed three times with ice-cold homogenization buffer. The sequences of the RIP-PCR primers are shown in Supplementary Table S1.

### Statistical analysis

Each experiment was performed at least three times. Data are shown as mean ± standard error (SE) and were analyzed using Student’s *t* test when appropriate. Differences were considered significant at *P* < 0.05.

## Results

### Symptoms of CECI in mice were alleviated after transplanting CD44^+^/CD105^+^ mAF-MSCs

**Fig. 1:**
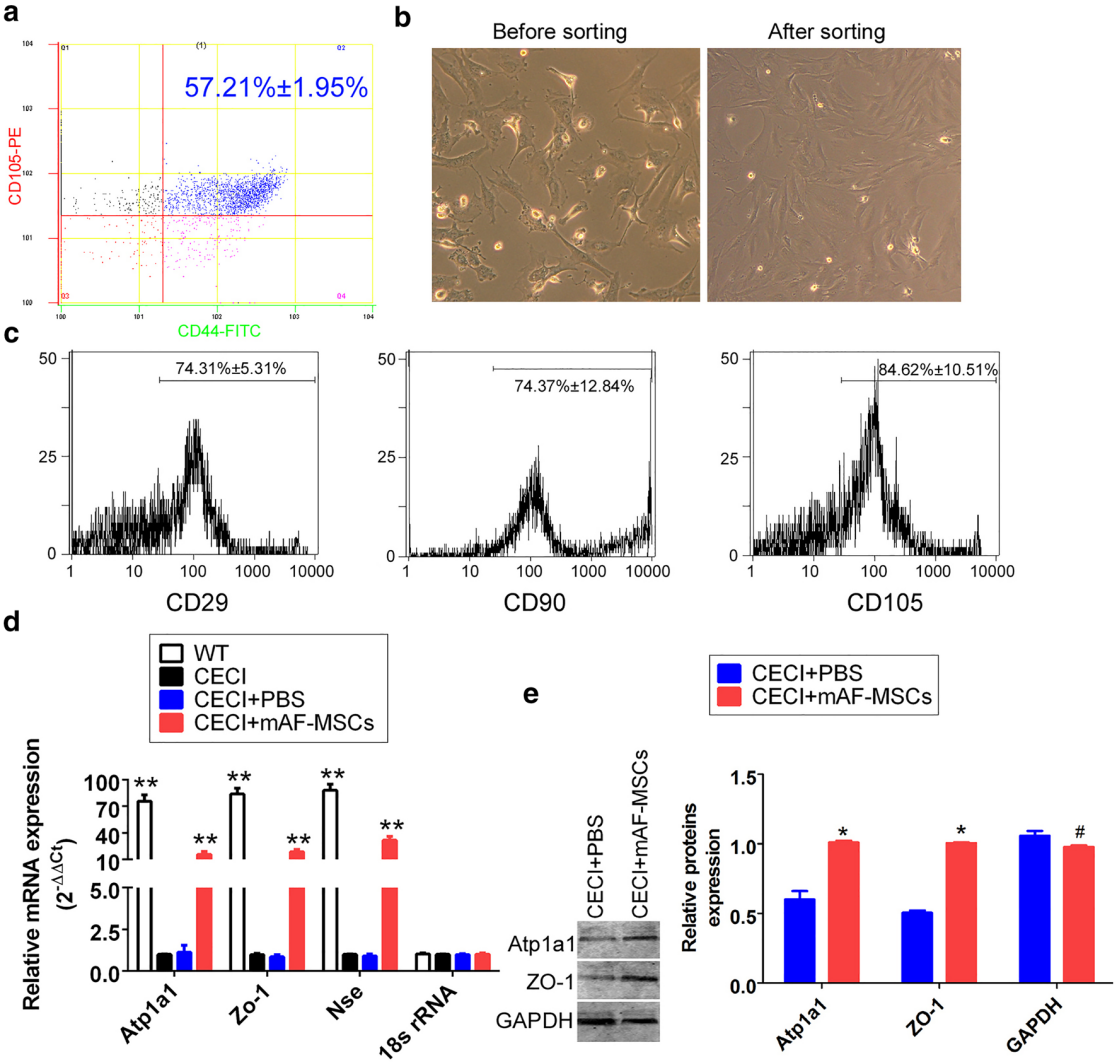
Symptoms of CECI in mice were alleviated after transplanting CD44 + /CD105 + mAF-MSCs. **a** Sorting results from FCM analysis. **b** Primary mAF-MSCs have similar phenotypes before and after sorting. Original magnification, × 200. **c** FCM analysis of mesenchymal stem cell marker expression in mAF-MSCs. Expression of ‘‘stemness’’ markers was high in mAF-MSCs. **d** qRT-PCR revealed that mRNA expression levels of *Atp1a1*, *Zo1*, and *Nse* were significantly higher in the mAF-MSCs transplant group than in the PBS group. **P < 0.01 vs. the PBS group; *t* test; *n* = 6. **e** Western blotting analysis revealed that the expression levels of both ATP1A1 and ZO-1 were elevated in the CECI mice in the mAF-MSCs transplant group as compared with those of the PBS transplant group. *P < 0.05 vs. PBS group; ^#^*P* > 0.05 vs. PBS group; *t* test; *n* = 6

To enrich the MSCs derived from mouse amniotic fluid, CD44 + /CD105 + mAF-MSCs were sorted from all mouse amniotic fluid using FCM (Fig. 1a). There was no significant difference in cell morphology before and after sorting, as revealed using bright field microscopy (Fig. 1b). The results of FCM analysis indicated that approximately 70% of the CD44^+^/CD105^+^ mAF-MSCs expressed the MSC biomarkers CD29, CD90, and CD105. This indicated that the CD44^+^/CD105^+^ mAF-MSCs possessed the “stemness” phenotype (Fig. 1c).

**Fig. 2:**
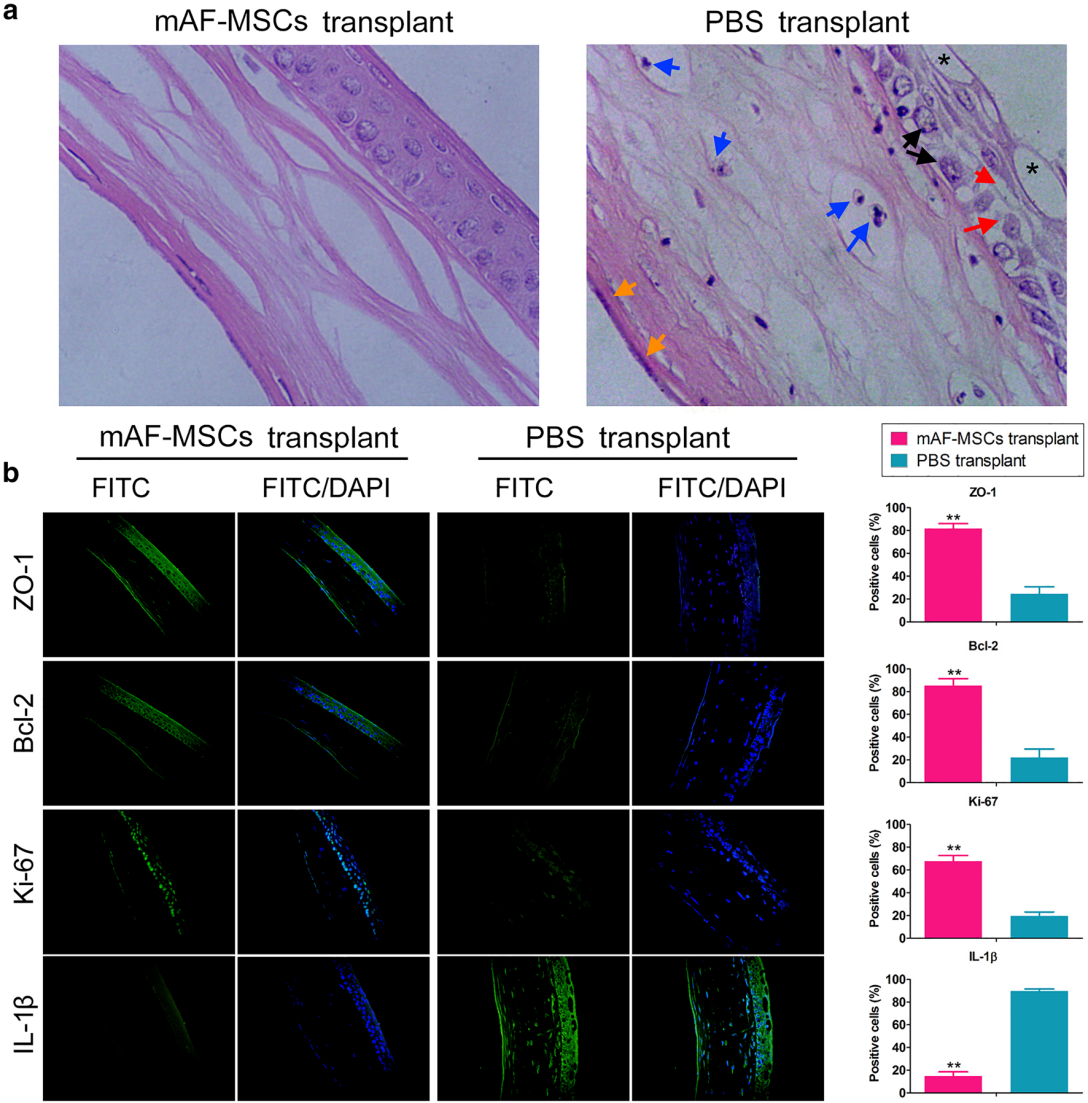
Pathology and identification of cell markers. **a** The results of HE staining. Magnification: × 400. The asterisk indicated that multiple vacuoles appeared in the outer layer of the cornea; the red arrow indicated that the connection between the corneal epithelial cells became very loose; the black arrow indicated that the nucleus of the corneal epithelial cells swelled and the whole cell showed necrotic structure; The blued arrow indicated that multiple inflammatory cells infiltrated in the middle layer of the cornea; The orange arrow indicated that the nucleus of corneal endothelium was swollen and the whole cell structure was fuzzy. **b** The results of immunofluorescence staining revealed that the ratio of KI67 + , ZO-1 + or BCL2 + cells were elevated significantly in the mAF-MSC-transplanted group compared with those in the PBS-transplanted group. *P < 0.05 vs. PBS group; *t* test; *n* = 6. Magnification: × 200

After 10 days of transplantation, the eyeballs of each group of mice were collected and used for qRT-PCR, western blotting analysis, and cDNA expression profile analysis. The mRNA expression levels of Na + -K + ATPase (*Atp1a1*), zonula occludens protein 1 (*Zo1*), and neuron specific enolase (*Nse*) in the mAF-MSC-transplanted group were significantly higher than those in the PBS group (Fig. 1d). A comparison of the mAF-MSC-transplanted and PBS-treated groups by western blotting demonstrated increased levels of both ATP1A1 and ZO-1 in the mAF-MSC-transplanted group (Fig. 1e, Supplementary Figure S1). In addition, result of HE staining showed that in the control group (PBS-treated group), the corneal epithelial cells showed significant edema and necrosis, inflammatory cell infiltration, and incomplete corneal morphology (multiple vacuoles appeared in the outer layer of the cornea (The asterisk on Fig. 2a); the connection between the corneal epithelial cells became very loose (The red arrow in Fig. 2a); the nucleus of the corneal epithelial cells swelled and the whole cell showed necrotic structure (The black arrow in Fig. 2a); and multiple inflammatory cells infiltrated in the middle layer of the cornea (The blue arrow in Fig. 2a). At the same time, the nucleus of corneal endothelium was swollen and the whole cell structure was fuzzy (The orange arrow in Fig. 2a).). However, in mAF-MSC-transplanted group, the infiltration of keratitis cells decreased significantly, the epithelial cells of the cornea were arranged completely, and the morphology was normal, which indicated that the symptoms of freezing injury had improved significantly (Fig. 2a). Meanwhile, the results of immunofluorescence staining revealed that the proportions of Ki67 + , ZO-1 + or Bcl-2 + cells were elevated significantly in mAF-MSC-transplanted group compared with those in the PBS-transplanted group (Fig. 2b). However, the proportions of interleukin 1 beta (IL-1β) + cells showed the opposite result (Fig. 2b). These results indicated that mAF-MSCs have the ability to promote the growth of corneal epithelial cells, reduce keratitis, and repair the corneal damage caused by low temperature.

### Promotion of RNA metabolic process and cell proliferation gene expression patterns in eyeballs transplanted with CD44^+^/CD105^+^ mAF-MSCs

**Fig. 3:**
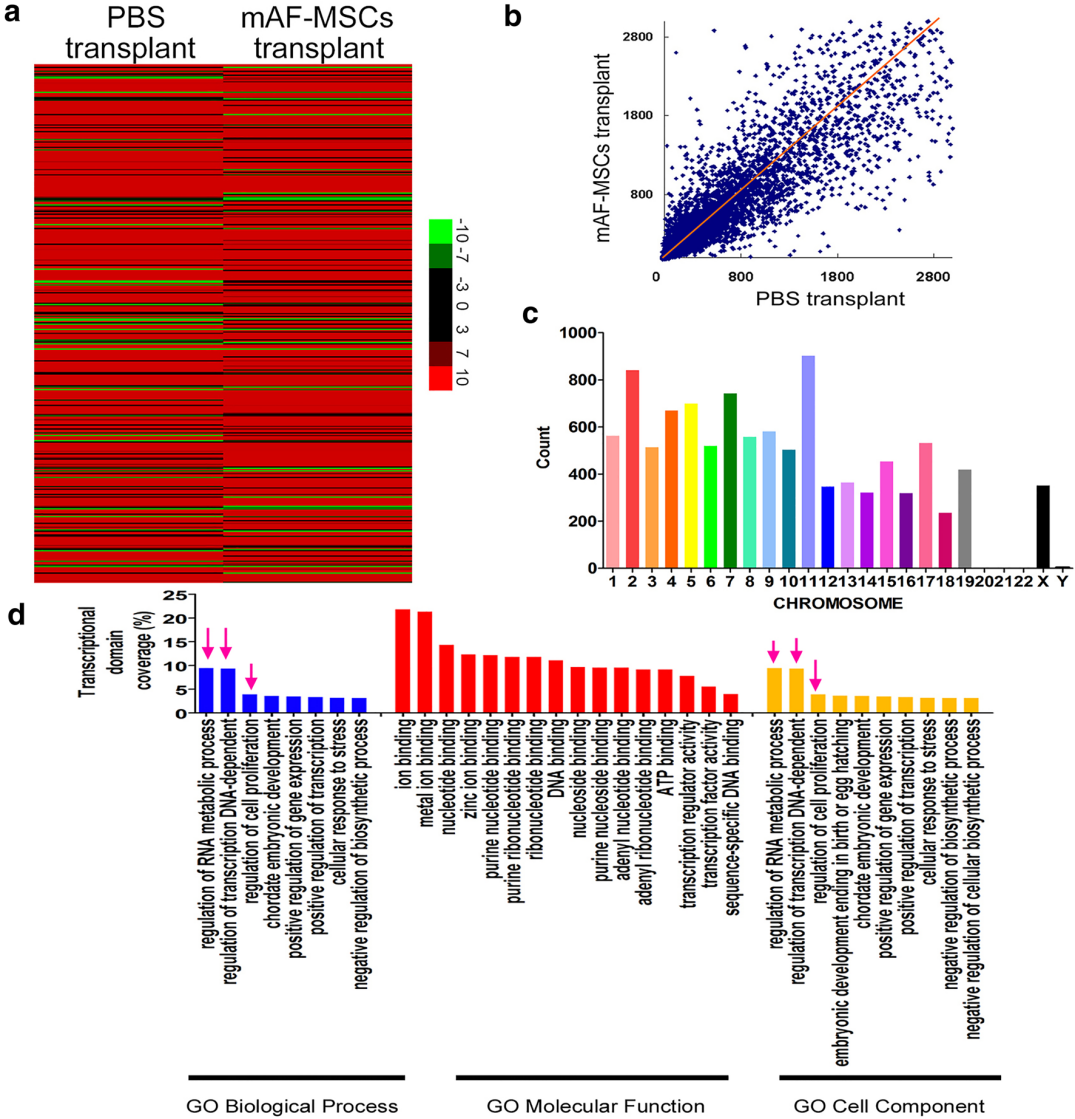
Microarray analysis of cDNA expression patterns in eyeballs transplanted with mAF-MSCs vs. PBS. **a** The clustering index of the microarray results revealed a number of genes that showed different expression levels between the mAF-MSCs transplant group and the PBS transplant group. **b** Scatter plot of the microarray results revealed a number of genes with different expression levels between the mAF-MSCs transplant group and the PBS transplant group. **c** Distribution of genes with significantly different expression levels on different chromosomes. **d** Different genes from the microarray defined by gene ontology (GO) analysis. Most of the biological processes were associated with the regulation of RNA metabolic process, cell proliferation, and gene expression

The clustering index and scatter plots of the cDNA microarray revealed a number of mRNAs with differential expression between the mAF-MSC-transplanted and PBS-treated groups (Fig. 3a). For the significance analysis of the microarray data, the fold change criterion (log_2_ (mAF-MSCs transplant/PBS transplant) ratio) of > 2.0 or <  − 2.0, and a *q* value < 0.01 were applied to identify significant differences. Based on these criteria, we identified 230 gene transcripts that differed in the mAF-MSC-transplanted vs. the PBS-treated groups (Fig. 3b, Supplementary Table S3). These differentially transcribed genes were distributed on chromosomes other than chromosomes 20, 21, and 22 (Fig. 3c). Gene Ontology (GO) is a well-known method to obtain data on gene annotations, and can represent the properties of the gene product. GO analysis was performed to annotate the differentially expressed genes from the microarray. Only terms with *P* < 0.01 were considered as having a significant predictive ability. The GO analysis indicated that a large number of differentially transcribed genes were concentrated in three process: Regulation of RNA metabolic processes, regulation of transcription in DNA-dependent processes, and regulation of cell proliferation (Fig. 3d, Supplementary Table S4). These results indicated that transplantation with CD44^+^/CD105^+^ mAF-MSCs altered the gene expression patterns in CECI mice eyeballs and promoted RNA metabolic process and cell proliferation.

### Promotion of mRNA N4-acetylcytidine (ac4C) modification and high expression of N-acetyltransferase in eyeballs transplanted with CD44^+^/CD105^+^ mAF-MSCs

**Fig. 4:**
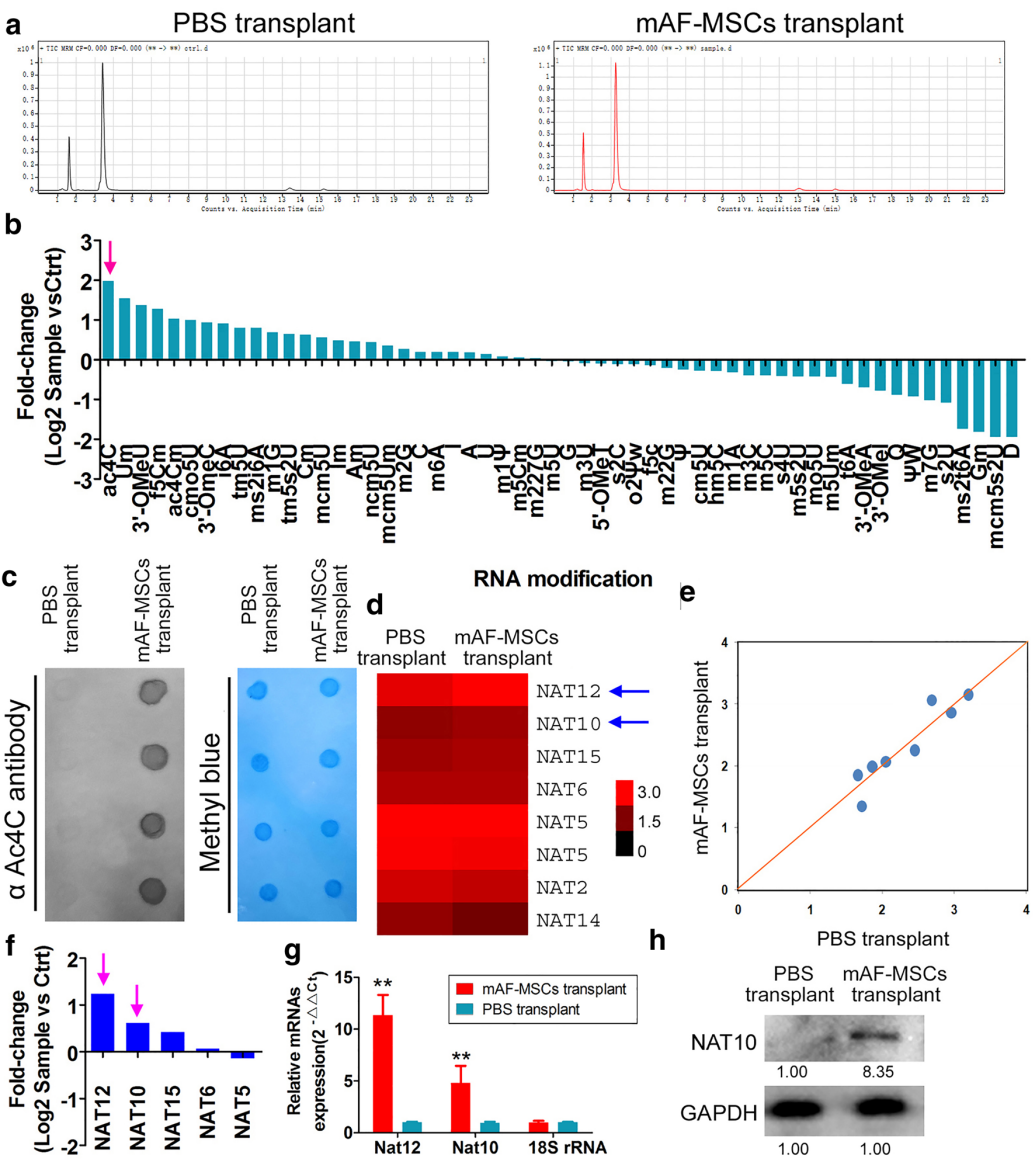
CD44 + /CD105 + mAF-MSCs promoted mRNA ac4C modification and high-expression of N-acetyltransferase in eyeballs. **a** HPLC–MS detection results showed that mRNA ac4C modification was significantly elevated in the mAF-MSCs transplant group. **b** The results of dot blotting indicated that the overall RNA ac4C level in the mAF-MSCs transplant group was significantly higher than that in the PBS transplant group. *P < 0.05 vs. PBS group; *t* test; *n* = 4. **c** The clustering index of the microarray results revealed that *N*-acetyltransferase expression differed between the two group. **d** The scatter plot of the microarray results revealed that *N*-acetyltransferase expression differed between the two groups. **e** QRT-PCR revealed that *Nat12* and *Nat10* mRNA levels were elevated significantly in mAF-MSC-transplanted mice. **P < 0.01 vs. PBS group; *t* test; *n* = 6. **f** Western blotting revealed that NAT10 protein levels were elevated significantly in mAF-MSC-transplanted mice. *P < 0.05 vs. PBS group; *t* test; n = 6

The HPLC–MS detection results showed that N4-acetylcytidine (ac4C) modification of mRNA was significantly elevated in the mAF-MSCs transplant group compared with that in the PBS transplant group (Fig. 4a, b, supplementary Table S4). Meanwhile, the results of dot blotting indicated that the overall RNA ac4C level in the mAF-MSCs transplant group was significantly higher than that in the PBS transplant group (Fig. 4c). The expression levels of genes encoding catalytic enzymes for the mRNA ac4C modification were analyzed. The clustering index and scatter plots of the cDNA microarray revealed that the expression levels of *Nat12*, *Nat10*, and *Nat15* were higher significantly the mAF-MSC-transplanted group than in the PBS-treated group (Fig. 4d–f). In addition, results of qRT-PCR and western blotting revealed that *Nat12*, *Nat10*, and *Nat15* mRNA and NAT10 protein levels were elevated significantly in the mAF-MSC-transplanted group compared with those in the PBS-treated group (Fig. 4g, h, Supplementary Figure S1). Thus, CD44 + /CD105 + mAF-MSCs could promote mRNA N4-acetylcytidine (ac4C) modification and the high expression of *N*-acetyltransferase in mouse eyeballs.

### CD44 + /CD105 + mAF-MSCs could promote ETV4/JUN/CCND2 signal axis activation and maintaining their stability in eyeballs

**Fig. 5:**
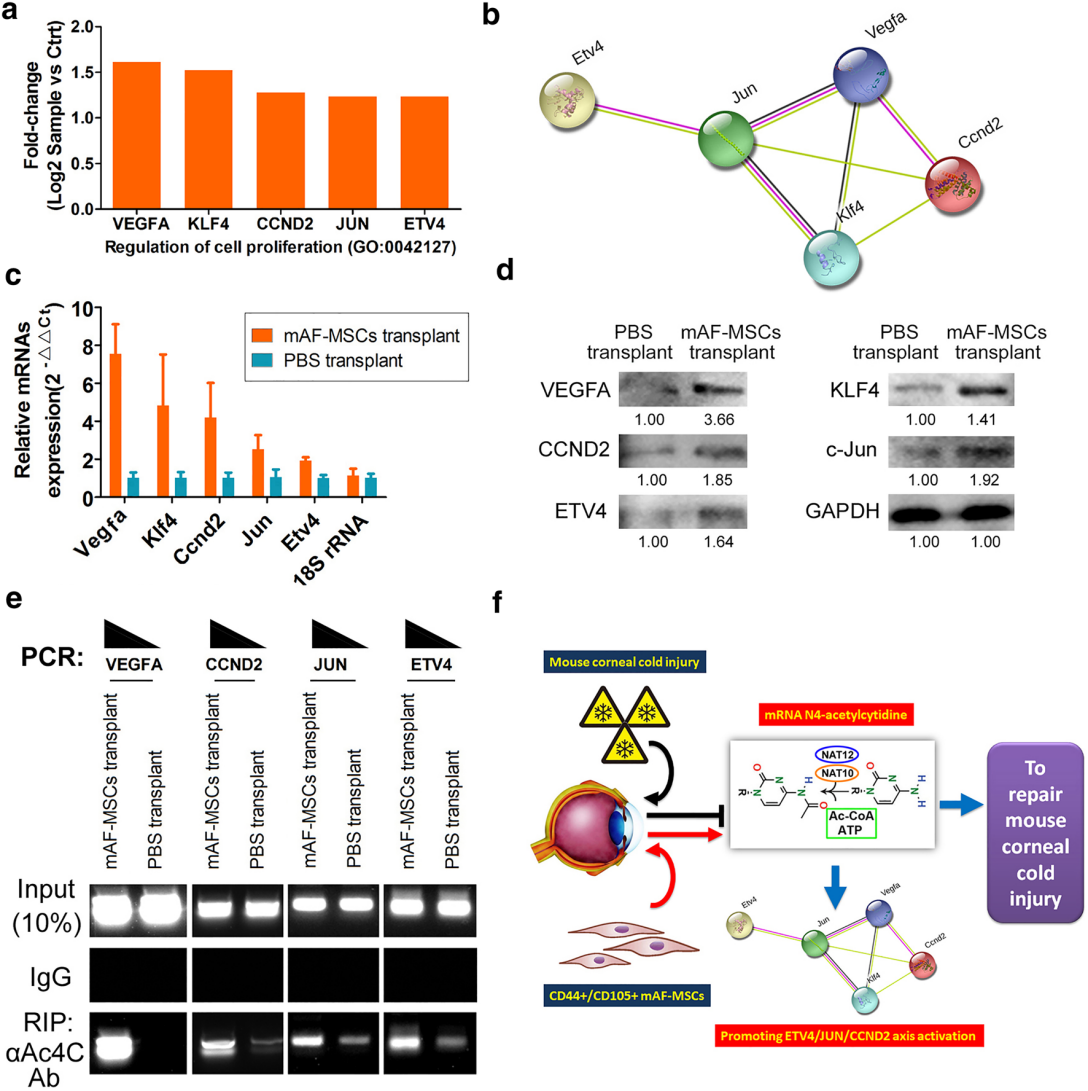
CD44 + /CD105 + mAF-MSCs promoted the activation of the ETV4/JUN/CCND2 signal axis and maintaining their stability. **a** The genes showing significantly high expression differences and that belonged to the above GO processes for regulation of cell proliferation were screened. **b** An interaction network comprising VEGFA, KLF4, CCND2, JUN, and ETV4 was identified using bioinformatic analysis (STRING: functional protein association networks). **c** QRT-PCR results showing that the expression levels of *Vegfa*, *Klf4*, *Ccnd2*, *Jun*, and *Etv4* were significantly higher in the mAF-MSCs-transplanted group than in the PBS-treated group. **P < 0.01 vs. PBS group; *P < 0.05 vs. PBS group; *t* test; *n* = 4. **d** Western blotting results showing that the levels of VEGFA, KLF4, CCND2, JUN, and ETV4 were significantly higher in the mAF-MSCs-transplanted group than in the PBS-treated group. *P < 0.05 vs. PBS group; *t* test; *n* = 6. **e** RIP-PCR results showing that a specific product of *Vegfa*, *Klf4*, *Ccnd2*, *Jun*, and *Etv4* mRNA specific coding region sites could be amplified using PCR from complexes from the mAF-MSC-transplanted group cross-linked using anti-ac4C antibodies (α ac4C ab). *P < 0.05 vs. PBS group; *t* test; *n* = 6. **f** The mechanism by which transplanted mAF-MSCs repair the mouse corneal cold injury comprises promotion of mRNA N4-acetylcytidine modification and ETV4/JUN/CCND2 signaling axis activation

Considering that the GO analysis indicated elevated regulation of cell proliferation in the mAF-MSC-transplanted group, we screened genes with significant high-expression differences that belonged to the above GO process (Fig. 5a). The interaction network involving vascular endothelial growth factor A (VEGFA), Kruppel like factor 4 (KLF4), cyclin D2 (CCND2), Jun N-terminal kinase (JUN), and ETS Variant Transcription Factor 4 ETV4 was identified using bioinformatic analysis (STRING: functional protein association networks; https://string-db.org/). Software predictions showed that ETV4 was intrinsically related to the proteins encoded by the four genes encoding VEGFA, KLF4, CCND2, and JUN. In addition, VEGFA, KLF4, CCND2, and JUN can form a completely closed signaling loop (Fig. 5b). QRT-PCR and western blotting showed that the expression levels of VEGFA, KLF4, CCND2, JUN, and ETV4 mRNA and protein in the mAF-MSC-transplanted group were significantly higher than those in the PBS-treated group (Fig. 5c, d, Supplementary Figure S1). In addition, RIP-PCR results showed that a specific product for *Vegfa*, *Klf4*, *Ccnd2*, *Jun*, and *Etv4* mRNA coding region sites could be amplified using PCR from complexes from the mAF-MSC-transplanted group cross-linked with the anti-ac4C antibody (α ac4C ab) (Fig. 5e). Very few of the above products were PCR amplified from complexes from the PBS-treated group cross-linked with the anti-ac4C antibody (Fig. 5e). Therefore, above results suggested that CD44 + /CD105 + mAF-MSCs could promote activation of the ETV4/JUN/CCND2 signaling axis and maintained their stability in eyeballs by stimulating the mRNA N4-acetylcytidine mRNA modification.

## Discussion

Amniotic fluid cells are commonly used for routine fetal genetic diagnosis of prenatal abnormalities [24, 25]. The amniotic fluid contains multiple types of cells derived from the developing fetus. These cells can differentiate into many different cell types such as adipose, muscle, bone, and neuronal cells [26, 27]. The CD44^+^/CD105^+^ subpopulation can be derived directly from amniotic fluid cells (AFCs) and cultured for ex vivo expansion [28–32]. Previous research has confirmed that CD44^+^/CD105^+^ cells have the ability to differentiate into a variety of cell types, including ectodermal, endodermal, mesodermal, hepatic cells, and cardiac muscle cells [29]. Notably, the CD44^+^/CD105^+^ cell subpopulation derived from AFCs could differentiate into cells of all three germ layers. Furthermore, our previous studies demonstrated that human AFCs (HuAFCs) express many different growth factors such as epidermal growth factor (EGF), basic fibroblast growth factor (bFGF), transforming growth factor alpha (TGF-α), transforming growth factor beta (TGF-β), and bone morphogenetic protein 4 (BMP-4), as well as the stem cell markers Nanog, octamer-binding protein 4 (OCT4), and Nestin. More importantly, amniotic fluid cells lack (major histocompatibility complex class II (MHC II) and express only low levels of MHC I [10, 12, 33, 34]. CD44^+^ HuAFCs can differentiate into dopaminergic neuron-like cells, which could improve behavioral recovery in a rat model of Parkinson's disease. In addition, pancreatic β-cell-like cells are also derived from CD44^+^/CD105^+^ HuAFCs [10, 12, 34]. In conclusion, AFCs are more easily acquired than other adult stem cells. There are many factors that could promote AFCs as an autologous transplantation source for stem cell therapy in the future. In the present study, when mAF-MSCs were transplanted into the damaged portion of the cornea after cryoinjury in mice, the mAF-MSCs were induced to proliferate and differentiate by the microenvironment (*Niche*). At the same time, mAF-MSCs also gradually repaired the corneal damage. We found that the expression levels of corneal endothelial cell markers increased significantly in the mAF-MSCs transplantation group, as was the expression level of Ki67. The results indicated there were new corneal endothelial cells in the mAF-MSCs transplantation group. However, in the control group, there was no significant increase in the expression of cellular proliferation factors. These results suggested that mAF-MSCs might stimulate the corneal microenvironment and lead to the proliferation of corneal endothelial cells.

Although the effect of mAF-MSCs treatment was clear, the next step was to reveal the mechanism of their effects. It has been reported that the proliferation and differentiation of stem cells or tumor cells are closely related to epigenetic modifications of RNA [13, 22]. Our HPLC/MS results showed that there were many significant differences in mRNA modification between the transplantation group and the control group, especially N4-acetylcytidine (ac4C). Some studies have reported that ac4C in mRNA is involved in the regulation of mRNA stability, processing, and translation [13, 15, 22]. NAT10 (a member of the N-acetyltransferase family) could improve mRNA stability and translation by maintaining mRNA acetylation to promote target gene expression [14, 22]. Accordingly, we found that expression levels of some *N*-acetyltransferases (NAT12, NAT10, and NAT15) were elevated significantly in the mAF-MSCs transplantation group compared with those in the PBS transplantation group. These results suggested that mAF-MSCs have the potential to enhance ac4C modification of mRNA via stimulating the expression *N*-acetyltransferases. Next, according to the results of the cDNA microArray and GO analysis, certain significantly differentially expressed genes (*Klf4*, *Ccnd2*, *Jun*, and *Etv4*) associated with regulation of cell proliferation were investigated. We found that the proteins encoded by these genes interacted with and stimulated each other. Further in-depth analysis showed that the mRNAs representing the above genes were significantly cross-linked using an anti-ac4C antibody in the mAF-MSCs transplantation group, indicating the occurrence of the ac4C modification on these mRNAs. The results correlated with those from the cDNA microarray, qPCR and western blotting experiments, which showed that the expression levels of members of the ETV4/JUN/CCND2 signaling axis were high in the mAF-MSCs transplantation group, indicating that their mRNAs were relatively stable. According to previous reports, ac4C modification of an mRNA could significantly improve its stability and maintain its expression [13–15, 17, 20, 22]. In this study, we also found that mAF-MSCs transplantation could significantly promote the expression of *N*-acyltransferases (NAT12, NAT10, and NAT15). Based on these data, we speculated that the activation and expression of the ETV4/JUN/CCND2 signal axis resulted from the ac4C modification of mRNA resulting from mAF-MSCs-mediated activation of members of the NAT family.

In conclusion, we found that mouse amniotic fluid mesenchymal stem cells could repair mouse corneal cold injury by promoting the activation of the ETV4/JUN/CCND2 signal axis and could improve its stability via stimulating N4-acetylcytidine modification of their mRNAs.

## Electronic supplementary material

Below is the link to the electronic supplementary material.

Supplementary file1 (DOC 34 kb)

Supplementary file2 (DOCX 20 kb)

Supplementary file3 (DOCX 37 kb)

Supplementary file4 (DOCX 19 kb)

Supplementary file5 (JPG 574 kb)
