# Supplementary material for: Amniotic fluid mesenchymal stem cells repair mouse corneal cold injury by promoting mRNA N4-acetylcytidine modification and ETV4/JUN/CCND2 signal axis activation
Source: Hum Cell. 2020 Oct 3;34(1):86–98. doi: 10.1007/s13577-020-00442-7 (PMC7788028; doi:10.1007/s13577-020-00442-7)
Supplement: Supplementary file 1 — Supplementary file1 (DOC 34 kb) [file 13577_2020_442_MOESM1_ESM.doc]

**Table 1 Primers for qRT-PCR**

| **Gene product** | **Forward (F) and reverse (R) primers (5’→3’)** |
| --- | --- |
| *Atp1a1* | F: GTATCGGAGCATGGTGACAAA  R: TCGTCCATAGACACTTCCTTCT |
| *Zo1* | F: GCTTTAGCGAACAGAAGGAGC  R: TTCATTTTTCCGAGACTTCACCA |
| *Nse* | F: GATGGGGACAAACAGCGTTAC  R: CACGGAGATACCTGAGCTGAT |
| *Nat12* | F: ATAGCCATGTTAGCCGTGGAC  R: CGTCACAGTCTCCTTCAACCA |
| *Nat10* | F: CACAAACATTCGCTACTGCTACT  R: AACGCTTCAAAATCCTGGAGG |
| *Vegfa* | F: GCACATAGAGAGAATGAGCTTCC  R: CTCCGCTCTGAACAAGGCT |
| *Klf4* | F: AGGAACTCTCTCACATGAAGCG  R: GGTCGTTGAACTCCTCGGTC |
| *Ccnd2* | F: TGAATTACCTGGACCGTTTCTTG  R: AGAGTTGTCGGTGTAAATGCAC |
| *Jun* | F: ACTCGGACCTTCTCACGTC  R: GGTCGGTGTAGTGGTGATGT |
| *Etv4* | F: CATTCCCAGATGATGTCTGCAT  R: CCACAGTTGTAAGGCACCCC |
| *18S rRNA* | F: AGGGGAGAGCGGGTAAGAGA  R: GGACAGGACTAGGCGGAACA |
