# Supplementary material for: Amniotic fluid mesenchymal stem cells repair mouse corneal cold injury by promoting mRNA N4-acetylcytidine modification and ETV4/JUN/CCND2 signal axis activation
Source: Hum Cell. 2020 Oct 3;34(1):86–98. doi: 10.1007/s13577-020-00442-7 (PMC7788028; doi:10.1007/s13577-020-00442-7)
Supplement: Supplementary file 2 — Supplementary file2 (DOCX 20 kb) [file 13577_2020_442_MOESM2_ESM.docx]

**Table S2. List of primary antibodies.**

| **Antibodies** | **Companies** | **Applications** |
| --- | --- | --- |
| Mouse anti-alpha 1 Sodium Potassium ATPase antibody [464.6] (ab7671) | Abcam, MA, USA | WB (1:1000) |
| Rabbit anti-ZO1 tight junction protein antibody (ab96587) | Abcam, MA, USA | WB (1:1000)  IF (1:300) |
| Rabbit anti-NAT10 antibody [EPR18663] (ab194297) | Abcam, MA, USA | WB (1:1000) |
| Rabbit anti-N4-acetylcytidine (ac4C) antibody [EPRNCI-184-128] (ab252215) | Abcam, MA, USA | RIP (1:100) |
| Rabbit anti-VEGFA antibody [EP1176Y] - C-terminal (ab52917) | Abcam, MA, USA | WB (1:1000) |
| Rabbit anti-Cyclin D2 antibody [EPR19659] (ab207604) | Abcam, MA, USA | WB (1:1000) |
| Rabbit anti-Pea3 (ETV4) antibody (ab189826) | Abcam, MA, USA | WB (1:1000) |
| Rabbit anti-KLF4 antibody [EPR19590] (ab215036) | Abcam, MA, USA | WB (1:1000) |
| Rabbit anti-c-Jun antibody [EP693Y] (ab40766) | Abcam, MA, USA | WB (1:1000) |
| Rabbit anti-Bcl-2 antibody [E17] (ab32124) | Abcam, MA, USA | IF (1:300) |
| Rabbit anti-Ki67 antibody (ab15580) | Abcam, MA, USA | IF (1:300) |
| Rabbit anti-IL-1 beta antibody [11E5] (ab8320) | Abcam, MA, USA | IF (1:300) |
| Rabbit anti-GAPDH antibody [EPR16891] (ab181602) | Abcam, MA, USA | WB (1:1000) |
| Goat anti-Rabbit IgG H&L (HRP) (ab97051) | Abcam, MA, USA | WB (1:1000) |
| Goat anti-Mouse IgG H&L (HRP) (ab6789) | Abcam, MA, USA | WB (1:1000) |
| Goat Anti-Rabbit IgG H&L (Alexa Fluor® 488) (ab150077) | Abcam, MA, USA | IF (1:300) |
