# Supplementary material for: Amniotic fluid mesenchymal stem cells repair mouse corneal cold injury by promoting mRNA N4-acetylcytidine modification and ETV4/JUN/CCND2 signal axis activation
Source: Hum Cell. 2020 Oct 3;34(1):86–98. doi: 10.1007/s13577-020-00442-7 (PMC7788028; doi:10.1007/s13577-020-00442-7)
Supplement: Supplementary file 3 — Supplementary file3 (DOCX 37 kb) [file 13577_2020_442_MOESM3_ESM.docx]

**Supplementary table 3. The differential gene transcripts of cDNA microArray**

| **PROBE_ID** | **ILMN_GENE** | **DIFFSCORE**  **(Log2 [mAF-MSCs transplant/PBS transplant]) > 2.0 or < -2.0)]** | **CHROMOSOME** | **DEFINITION** |
| --- | --- | --- | --- | --- |
| ILMN_2617468 | CHAC1 | 6.28 | 2 | Mus musculus ChaC, cation transport regulator-like 1 (E. coli) (Chac1), mRNA. |
| ILMN_2948143 | SLC7A11 | 5.72 | 3 | Mus musculus solute carrier family 7 (cationic amino acid transporter, y+ system), member 11 (Slc7a11), mRNA. |
| ILMN_1225528 | TRIB3 | 5.21 | 2 | Mus musculus tribbles homolog 3 (Drosophila) (Trib3), mRNA. |
| ILMN_2661650 | REEP6 | 4.40 | 10 | Mus musculus receptor accessory protein 6 (Reep6), mRNA. |
| ILMN_2733193 | CTH | 4.34 | 3 | Mus musculus cystathionase (cystathionine gamma-lyase) (Cth), mRNA. |
| ILMN_3162820 | ODZ4 | 4.12 | 7 | Mus musculus odd Oz/ten-m homolog 4 (Drosophila) (Odz4), mRNA. |
| ILMN_3102736 | GM129 | 3.81 | 3 | Mus musculus gene model 129, (NCBI) (Gm129), mRNA. XM_907670 XM_920513 XM_920520 XM_920527 XM_920533 |
| ILMN_2977492 | MOXD1 | 3.79 | 10 | Mus musculus monooxygenase, DBH-like 1 (Moxd1), mRNA. |
| ILMN_3111575 | MEF2B | 3.79 | 8 | Mus musculus myocyte enhancer factor 2B (Mef2b), transcript variant 2, mRNA. |
| ILMN_2947559 | RBM35A | 3.61 | 4 | Mus musculus RNA binding motif protein 35A (Rbm35a), mRNA. |
| ILMN_2745614 | FAM134B | 3.60 | 15 | Mus musculus family with sequence similarity 134, member B (Fam134b), transcript variant 2, mRNA. |
| ILMN_2491392 | TM7SF3 | 3.54 | 6 | Mus musculus transmembrane 7 superfamily member 3 (Tm7sf3), mRNA. |
| ILMN_2783225 | TCFAP2C | 3.53 | 2 | Mus musculus transcription factor AP-2, gamma (Tcfap2c), mRNA. |
| ILMN_3126646 | GRIP1 | 3.44 | 10 | Mus musculus glutamate receptor interacting protein 1 (Grip1), transcript variant 2, mRNA. |
| ILMN_2590520 | H2-BL | 3.44 | 17 | Mus musculus histocompatibility 2, blastocyst (H2-Bl), mRNA. |
| ILMN_3029489 | GM129 | 3.40 | 3 | Mus musculus gene model 129, (NCBI) (Gm129), mRNA. XM_907670 XM_920513 XM_920520 XM_920527 XM_920533 |
| ILMN_2898917 | OTUB2 | 3.31 | 12 | Mus musculus OTU domain, ubiquitin aldehyde binding 2 (Otub2), mRNA. |
| ILMN_1254295 | SOX21 | 3.27 | 14 | Mus musculus SRY-box containing gene 21 (Sox21), mRNA. |
| ILMN_2712075 | LCN2 | 3.25 | 2 | Mus musculus lipocalin 2 (Lcn2), mRNA. |
| ILMN_2987637 | FBXO15 | 3.24 | 18 | Mus musculus F-box protein 15 (Fbxo15), mRNA. |
| ILMN_2990152 | DCLK2 | 3.23 | 3 | Mus musculus doublecortin-like kinase 2 (Dclk2), mRNA. |
| ILMN_2603334 | HOXA1 | 3.19 | 6 | Mus musculus homeo box A1 (Hoxa1), mRNA. |
| ILMN_2907370 | OPTN | 3.15 | 2 | Mus musculus optineurin (Optn), mRNA. |
| ILMN_2976159 | MREG | 3.10 | 1 | Mus musculus melanoregulin (Mreg), mRNA. |
| ILMN_1254358 | IGFBP5 | 3.09 | 1 | Mus musculus insulin-like growth factor binding protein 5 (Igfbp5), mRNA. |
| ILMN_2703079 | PUNC | 3.08 | 9 | Mus musculus putative neuronal cell adhesion molecule (Punc), mRNA. |
| ILMN_2739544 | STC2 | 3.07 | 11 | Mus musculus stanniocalcin 2 (Stc2), mRNA. |
| ILMN_2887630 | FABP3 | 3.07 | 4 | Mus musculus fatty acid binding protein 3, muscle and heart (Fabp3), mRNA. |
| ILMN_2919860 | CDS1 | 3.06 | 5 | Mus musculus CDP-diacylglycerol synthase 1 (Cds1), mRNA. |
| ILMN_1237338 | DDX25 | 3.05 | 9 | Mus musculus DEAD (Asp-Glu-Ala-Asp) box polypeptide 25 (Ddx25), mRNA. |
| ILMN_2840856 | RIPK4 | 3.04 | 16 | Mus musculus receptor-interacting serine-threonine kinase 4 (Ripk4), mRNA. |
| ILMN_2501605 | PRKCB | 3.04 | 7 | Mus musculus protein kinase C, beta (Prkcb), mRNA. |
| ILMN_2632092 | PPP1R9A | 2.98 | 6 | Mus musculus protein phosphatase 1, regulatory (inhibitor) subunit 9A (Ppp1r9a), mRNA. |
| ILMN_1260046 | SFT2D2 | 2.94 | 1 | Mus musculus SFT2 domain containing 2 (Sft2d2), mRNA. |
| ILMN_2648967 | ARRDC4 | 2.90 | 7 | Mus musculus arrestin domain containing 4 (Arrdc4), transcript variant 1, mRNA. |
| ILMN_2652909 | DDIT3 | 2.90 | 10 | Mus musculus DNA-damage inducible transcript 3 (Ddit3), mRNA. |
| ILMN_2676606 | OPTN | 2.90 | 2 | Mus musculus optineurin (Optn), mRNA. |
| ILMN_3086136 | AGAP1 | 2.90 | 1 | Mus musculus ArfGAP with GTPase domain, ankyrin repeat and PH domain 1 (Agap1), transcript variant 2, mRNA. |
| ILMN_1234990 | GAP43 | 2.89 | 16 | Mus musculus growth associated protein 43 (Gap43), mRNA. |
| ILMN_1225085 | PRKCB | 2.89 | 7 | Mus musculus protein kinase C, beta (Prkcb), mRNA. |
| ILMN_2867076 | H2-OA | 2.88 | 17 | Mus musculus histocompatibility 2, O region alpha locus (H2-Oa), mRNA. |
| ILMN_2703698 | TDRKH | 2.86 | 3 | Mus musculus tudor and KH domain containing protein (Tdrkh), mRNA. |
| ILMN_3077377 | WARS | 2.85 | 12 | Mus musculus tryptophanyl-tRNA synthetase (Wars), mRNA. |
| ILMN_1213378 | LONRF3 | 2.82 | X | Mus musculus LON peptidase N-terminal domain and ring finger 3 (Lonrf3), mRNA. |
| ILMN_1239134 | LYPD1 | 2.82 | 1 | Mus musculus Ly6/Plaur domain containing 1 (Lypd1), mRNA. |
| ILMN_1257987 | SLC7A3 | 2.81 | X | Mus musculus solute carrier family 7 (cationic amino acid transporter, y+ system), member 3 (Slc7a3), mRNA. |
| ILMN_1224637 | FOXQ1 | 2.79 | 13 | Mus musculus forkhead box Q1 (Foxq1), mRNA. |
| ILMN_2669714 | CTSA | 2.77 | 2 | Mus musculus cathepsin A (Ctsa), transcript variant 2, mRNA. |
| ILMN_3112873 | TXNIP | 2.72 | 3 | Mus musculus thioredoxin interacting protein (Txnip), transcript variant 1, mRNA. |
| ILMN_2927373 | DDIT3 | 2.72 | 10 | Mus musculus DNA-damage inducible transcript 3 (Ddit3), mRNA. |
| ILMN_2691752 | OTX2 | 2.71 | 14 | Mus musculus orthodenticle homolog 2 (Drosophila) (Otx2), mRNA. |
| ILMN_2734624 | TDRKH | 2.68 | 3 | Mus musculus tudor and KH domain containing protein (Tdrkh), mRNA. |
| ILMN_1246289 | GOT1L1 | 2.68 | 8 | Mus musculus glutamic-oxaloacetic transaminase 1-like 1 (Got1l1), mRNA. |
| ILMN_2898319 | ALDH1L2 | 2.65 | 10 | Mus musculus aldehyde dehydrogenase 1 family, member L2 (Aldh1l2), mRNA. |
| ILMN_2662926 | EGR1 | 2.65 | 18 | Mus musculus early growth response 1 (Egr1), mRNA. |
| ILMN_2606470 | CLIP4 | 2.62 | 17 | Mus musculus CAP-GLY domain containing linker protein family, member 4 (Clip4), mRNA. |
| ILMN_2964324 | IGFBP5 | 2.62 | 1 | Mus musculus insulin-like growth factor binding protein 5 (Igfbp5), mRNA. |
| ILMN_2806720 | RBKS | 2.62 | 5 | Mus musculus ribokinase (Rbks), mRNA. |
| ILMN_3006123 | ASNS | 2.62 | 6 | Mus musculus asparagine synthetase (Asns), mRNA. |
| ILMN_1238592 | PIGH | 2.59 | 12 | Mus musculus phosphatidylinositol glycan anchor biosynthesis, class H (Pigh), mRNA. |
| ILMN_2591781 | TMEM49 | 2.58 | 11 | Mus musculus transmembrane protein 49 (Tmem49), mRNA. |
| ILMN_1248657 | IL17RD | 2.57 | 14 | Mus musculus interleukin 17 receptor D (Il17rd), mRNA. |
| ILMN_1218934 | RDM1 | 2.57 | 11 | Mus musculus RAD52 motif 1 (Rdm1), mRNA. |
| ILMN_1222471 | GMFG | 2.52 | 7 | Mus musculus glia maturation factor, gamma (Gmfg), transcript variant 1, mRNA. |
| ILMN_2911307 | GM347 | 2.50 | 2 | Mus musculus gene model 347, (NCBI) (Gm347), mRNA. |
| ILMN_2697615 | MAPK12 | 2.49 | 15 | Mus musculus mitogen-activated protein kinase 12 (Mapk12), mRNA. |
| ILMN_1243370 | CYB5R1 | 2.49 | 1 | Mus musculus cytochrome b5 reductase 1 (Cyb5r1), mRNA. |
| ILMN_1232186 | UNG | 2.48 | 5 | Mus musculus uracil DNA glycosylase (Ung), transcript variant 2, mRNA. |
| ILMN_1213609 | TXNIP | 2.47 | 3 | Mus musculus thioredoxin interacting protein (Txnip), transcript variant 2, mRNA. |
| ILMN_2440242 | ZIC3 | 2.47 | X | Mus musculus zinc finger protein of the cerebellum 3 (Zic3), mRNA. |
| ILMN_2667384 | SLC6A9 | 2.46 | 4 | Mus musculus solute carrier family 6 (neurotransmitter transporter, glycine), member 9 (Slc6a9), mRNA. |
| ILMN_1227907 | GMFG | 2.46 | 7 | Mus musculus glia maturation factor, gamma (Gmfg), transcript variant 1, mRNA. |
| ILMN_3115796 | CD40 | 2.46 | 2 | Mus musculus CD40 antigen (Cd40), transcript variant 5, mRNA. |
| ILMN_2525423 | WWC1 | 2.44 | 11 | Mus musculus WW, C2 and coiled-coil domain containing 1 (Wwc1), mRNA. |
| ILMN_3093626 | PORCN | 2.44 | X | Mus musculus porcupine homolog (Drosophila) (Porcn), transcript variant Mporc-b, mRNA. |
| ILMN_1258935 | SULT4A1 | 2.44 | 15 | Mus musculus sulfotransferase family 4A, member 1 (Sult4a1), mRNA. |
| ILMN_2757008 | ALDH1L2 | 2.42 | 10 | Mus musculus aldehyde dehydrogenase 1 family, member L2 (Aldh1l2), mRNA. |
| ILMN_2959293 | UPP1 | 2.41 | 11 | Mus musculus uridine phosphorylase 1 (Upp1), mRNA. |
| ILMN_2878548 | MTHFD2 | 2.41 | 6 | Mus musculus methylenetetrahydrofolate dehydrogenase (NAD+ dependent), methenyltetrahydrofolate cyclohydrolase (Mthfd2), mRNA. |
| ILMN_1228748 | FOXA3 | 2.41 | 7 | Mus musculus forkhead box A3 (Foxa3), mRNA. |
| ILMN_2959292 | UPP1 | 2.40 | 11 | Mus musculus uridine phosphorylase 1 (Upp1), mRNA. |
| ILMN_3144984 | ARL4A | 2.39 | 12 | Mus musculus ADP-ribosylation factor-like 4A (Arl4a), transcript variant 1, mRNA. |
| ILMN_2742152 | GADD45A | 2.37 | 6 | Mus musculus growth arrest and DNA-damage-inducible 45 alpha (Gadd45a), mRNA. |
| ILMN_2943270 | LRRN4CL | 2.35 | 19 | Mus musculus LRRN4 C-terminal like (Lrrn4cl), mRNA. |
| ILMN_3156343 | WARS | 2.35 | 12 | Mus musculus tryptophanyl-tRNA synthetase (Wars), mRNA. |
| ILMN_1252545 | MTAP7 | 2.35 | 10 | Mus musculus microtubule-associated protein 7 (Mtap7), mRNA. |
| ILMN_1256771 | ADRBK2 | 2.35 | 5 | Mus musculus adrenergic receptor kinase, beta 2 (Adrbk2), transcript variant 2, mRNA. |
| ILMN_2947234 | CBX7 | 2.33 | 15 | Mus musculus chromobox homolog 7 (Cbx7), mRNA. |
| ILMN_2643159 | SIRT1 | 2.33 | 10 | Mus musculus sirtuin 1 (silent mating type information regulation 2, homolog) 1 (S. cerevisiae) (Sirt1), mRNA. |
| ILMN_2874270 | HOXA2 | 2.33 | 6 | Mus musculus homeo box A2 (Hoxa2), mRNA. |
| ILMN_2801427 | CMTM8 | 2.32 | 9 | Mus musculus CKLF-like MARVEL transmembrane domain containing 8 (Cmtm8), mRNA. |
| ILMN_1247947 | SQRDL | 2.29 | 2 | Mus musculus sulfide quinone reductase-like (yeast) (Sqrdl), mRNA. |
| ILMN_2619639 | AI428936 | 2.29 | 7 | Mus musculus expressed sequence AI428936 (AI428936), mRNA. |
| ILMN_1241345 | PRRG3 | 2.29 | X | Mus musculus proline rich Gla (G-carboxyglutamic acid) 3 (transmembrane) (Prrg3), mRNA. |
| ILMN_2622190 | PNMA2 | 2.27 | 14 | Mus musculus paraneoplastic antigen MA2 (Pnma2), mRNA. |
| ILMN_3001481 | CHRNB1 | 2.27 | 11 | Mus musculus cholinergic receptor, nicotinic, beta polypeptide 1 (muscle) (Chrnb1), mRNA. |
| ILMN_2660596 | RUNDC3B | 2.27 | 5 | Mus musculus RUN domain containing 3B (Rundc3b), mRNA. |
| ILMN_2515601 | VLDLR | 2.26 | 19 | Mus musculus very low density lipoprotein receptor (Vldlr), mRNA. |
| ILMN_2888552 | SLC1A4 | 2.25 | 11 | Mus musculus solute carrier family 1 (glutamate/neutral amino acid transporter), member 4 (Slc1a4), mRNA. |
| ILMN_2943176 | KLHL8 | 2.25 | 5 | Mus musculus kelch-like 8 (Drosophila) (Klhl8), mRNA. |
| ILMN_2721198 | GGNBP1 | 2.24 | 17 | Mus musculus gametogenetin binding protein 1 (Ggnbp1), mRNA. |
| ILMN_2718030 | ANK2 | 2.22 | 3 | Mus musculus ankyrin 2, brain (Ank2), transcript variant 3, mRNA. |
| ILMN_2433946 | GLCE | 2.22 | 9 | Mus musculus glucuronyl C5-epimerase (Glce), mRNA. |
| ILMN_1248537 | DUSP6 | 2.21 | 10 | Mus musculus dual specificity phosphatase 6 (Dusp6), mRNA. |
| ILMN_1237186 | SPINT1 | 2.21 | 2 | Mus musculus serine protease inhibitor, Kunitz type 1 (Spint1), mRNA. |
| ILMN_1250507 | ELOVL4 | 2.21 | 9 | Mus musculus elongation of very long chain fatty acids (FEN1/Elo2, SUR4/Elo3, yeast)-like 4 (Elovl4), mRNA. |
| ILMN_2807016 | TSPAN7 | 2.19 | X | Mus musculus tetraspanin 7 (Tspan7), mRNA. |
| ILMN_2595359 | SLC3A2 | 2.19 | 19 | Mus musculus solute carrier family 3 (activators of dibasic and neutral amino acid transport), member 2 (Slc3a2), mRNA. |
| ILMN_1221161 | ZFP57 | 2.19 | 17 | Mus musculus zinc finger protein 57 (Zfp57), transcript variant 1, mRNA. |
| ILMN_1219154 | MT2 | 2.19 | 8 | Mus musculus metallothionein 2 (Mt2), mRNA. |
| ILMN_2947568 | GADD45A | 2.18 | 6 | Mus musculus growth arrest and DNA-damage-inducible 45 alpha (Gadd45a), mRNA. |
| ILMN_1215908 | CHD7 | 2.18 | 4 | Mus musculus chromodomain helicase DNA binding protein 7 (Chd7), mRNA. |
| ILMN_3124235 | SALL4 | 2.17 | 2 | Mus musculus sal-like 4 (Drosophila) (Sall4), transcript variant b, mRNA. |
| ILMN_1241838 | CACNA1G | 2.17 | 11 | Mus musculus calcium channel, voltage-dependent, T type, alpha 1G subunit (Cacna1g), mRNA. |
| ILMN_1228091 | BCOR | 2.16 | X | Mus musculus BCL6 interacting corepressor (Bcor), transcript variant c, mRNA. |
| ILMN_2688533 | RIBC1 | 2.16 | X | Mus musculus RIB43A domain with coiled-coils 1 (Ribc1), mRNA. |
| ILMN_2745889 | ACOT2 | 2.14 | 12 | Mus musculus acyl-CoA thioesterase 2 (Acot2), nuclear gene encoding mitochondrial protein, mRNA. |
| ILMN_3075774 | CTDSPL2 | 2.14 | 2 | Mus musculus CTD (carboxy-terminal domain, RNA polymerase II, polypeptide A) small phosphatase like 2 (Ctdspl2), mRNA. |
| ILMN_2536776 | E130308A19RIK | 2.14 | 4 | Mus musculus RIKEN cDNA E130308A19 gene (E130308A19Rik), transcript variant 2, mRNA. |
| ILMN_2502290 | TRIM71 | 2.14 | 9 | Mus musculus tripartite motif-containing 71 (Trim71), mRNA. |
| ILMN_2857114 | STRBP | 2.13 | 2 | Mus musculus spermatid perinuclear RNA binding protein (Strbp), mRNA. |
| ILMN_2703023 | FCHO1 | 2.13 | 8 | Mus musculus FCH domain only 1 (Fcho1), mRNA. |
| ILMN_3096287 | BCOR | 2.12 | X | Mus musculus Bcl6 interacting corepressor (Bcor), transcript variant a, mRNA. |
| ILMN_1218264 | VLDLR | 2.11 | 19 | Mus musculus very low density lipoprotein receptor (Vldlr), mRNA. |
| ILMN_1214703 | NME7 | 2.11 | 1 | Mus musculus non-metastatic cells 7, protein expressed in (nucleoside-diphosphate kinase) (Nme7), transcript variant 1, mRNA. |
| ILMN_1240318 | SLC7A7 | 2.11 | 14 | Mus musculus solute carrier family 7 (cationic amino acid transporter, y+ system), member 7 (Slc7a7), mRNA. |
| ILMN_2735522 | LRP2 | 2.10 | 2 | Mus musculus low density lipoprotein receptor-related protein 2 (Lrp2), mRNA. |
| ILMN_1249046 | CMTM8 | 2.09 | 9 | Mus musculus CKLF-like MARVEL transmembrane domain containing 8 (Cmtm8), mRNA. |
| ILMN_2925711 | DUSP6 | 2.09 | 10 | Mus musculus dual specificity phosphatase 6 (Dusp6), mRNA. |
| ILMN_2677332 | HIC2 | 2.09 | 16 | Mus musculus hypermethylated in cancer 2 (Hic2), mRNA. |
| ILMN_2796472 | VLDLR | 2.08 | 19 | Mus musculus very low density lipoprotein receptor (Vldlr), mRNA. |
| ILMN_3072117 | RIC3 | 2.06 | 7 | Mus musculus resistance to inhibitors of cholinesterase 3 homolog (C. elegans) (Ric3), transcript variant 1, mRNA. |
| ILMN_1257801 | GPR19 | 2.06 | 6 | Mus musculus G protein-coupled receptor 19 (Gpr19), mRNA. |
| ILMN_2959291 | UPP1 | 2.06 | 11 | Mus musculus uridine phosphorylase 1 (Upp1), mRNA. |
| ILMN_2625279 | PACRG | 2.05 | 17 | Mus musculus Park2 co-regulated (Pacrg), mRNA. |
| ILMN_2759335 | RNMT | 2.05 | 18 | Mus musculus RNA (guanine-7-) methyltransferase (Rnmt), mRNA. |
| ILMN_3070389 | ENSMUSG00000068790 | 2.05 | 14 | Mus musculus predicted gene, ENSMUSG00000068790 (ENSMUSG00000068790), mRNA. |
| ILMN_2860616 | HIST1H3H | 2.04 | 13 | Mus musculus histone cluster 1, H3h (Hist1h3h), mRNA. |
| ILMN_2948945 | SESN2 | 2.04 | 4 | Mus musculus sestrin 2 (Sesn2), mRNA. |
| ILMN_3143604 | GNG2 | 2.04 | 14 | Mus musculus guanine nucleotide binding protein (G protein), gamma 2 (Gng2), transcript variant 2, mRNA. |
| ILMN_3094023 | PSMF1 | 2.03 | 2 | Mus musculus proteasome (prosome, macropain) inhibitor subunit 1 (Psmf1), transcript variant 2, mRNA. |
| ILMN_2591777 | TMEM49 | 2.03 | 11 | Mus musculus transmembrane protein 49 (Tmem49), mRNA. |
| ILMN_3028637 | CALCA | 2.03 | 7 | Mus musculus calcitonin/calcitonin-related polypeptide, alpha (Calca), transcript variant 2, mRNA. |
| ILMN_2898886 | OTTMUSG00000010673 | 2.02 | 4 | Mus musculus predicted gene, OTTMUSG00000010673 (OTTMUSG00000010673), mRNA. XM_902030 XM_902032 XM_902035 XM_918501 |
| ILMN_1220360 | UNC13B | 2.02 | 4 | Mus musculus unc-13 homolog B (C. elegans) (Unc13b), mRNA. |
| ILMN_2595478 | SALL4 | 2.02 | 2 | Mus musculus sal-like 4 (Drosophila) (Sall4), transcript variant a, mRNA. |
| ILMN_3095356 | LOC545013 | 2.02 | 14 | Mus musculus hypothetical protein LOC545013 (LOC545013), mRNA. |
| ILMN_1230281 | OPHN1 | 2.02 | X | Mus musculus oligophrenin 1 (Ophn1), mRNA. |
| ILMN_2513525 | LIN28B | 2.01 | 10 | Mus musculus lin-28 homolog B (C. elegans) (Lin28b), mRNA. |
| ILMN_2501719 | USH1C | 2.01 | 7 | Mus musculus Usher syndrome 1C homolog (human) (Ush1c), transcript variant b3, mRNA. |
| ILMN_1238558 | ARID3B | 2.01 | 9 | Mus musculus AT rich interactive domain 3B (BRIGHT-like) (Arid3b), mRNA. |
| ILMN_2618302 | HOXA2 | 2.01 | 6 | Mus musculus homeo box A2 (Hoxa2), mRNA. |
| ILMN_2589318 | POU6F1 | 2.00 | 15 | Mus musculus POU domain, class 6, transcription factor 1 (Pou6f1), mRNA. |
| ILMN_2445848 | ZFP238 | 2.00 | 1 | Mus musculus zinc finger protein 238 (Zfp238), transcript variant 2, mRNA. |
| ILMN_1257551 | CRISPLD2 | -2.00 | 8 | Mus musculus cysteine-rich secretory protein LCCL domain containing 2 (Crispld2), mRNA. |
| ILMN_2730797 | SLC25A10 | -2.01 | 11 | Mus musculus solute carrier family 25 (mitochondrial carrier, dicarboxylate transporter), member 10 (Slc25a10), nuclear gene encoding mitochondrial protein, mRNA. |
| ILMN_1224619 | ST3GAL1 | -2.02 | 15 | Mus musculus ST3 beta-galactoside alpha-2,3-sialyltransferase 1 (St3gal1), mRNA. |
| ILMN_1219253 | OLFML1 | -2.02 | 7 | Mus musculus olfactomedin-like 1 (Olfml1), mRNA. |
| ILMN_2788036 | ACSBG1 | -2.02 | 9 | Mus musculus acyl-CoA synthetase bubblegum family member 1 (Acsbg1), mRNA. |
| ILMN_2594525 | NSDHL | -2.02 | X | Mus musculus NAD(P) dependent steroid dehydrogenase-like (Nsdhl), mRNA. |
| ILMN_3160067 | LHX9 | -2.03 | 1 | Mus musculus LIM homeobox protein 9 (Lhx9), transcript variant 1, mRNA. |
| ILMN_3162239 | PRR7 | -2.04 | 13 | Mus musculus proline rich 7 (synaptic) (Prr7), mRNA. |
| ILMN_2903972 | PDGFRB | -2.04 | 18 | Mus musculus platelet derived growth factor receptor, beta polypeptide (Pdgfrb), mRNA. |
| ILMN_2958207 | NSDHL | -2.05 | X | Mus musculus NAD(P) dependent steroid dehydrogenase-like (Nsdhl), mRNA. |
| ILMN_2955694 | SPAG1 | -2.06 | 15 | Mus musculus sperm associated antigen 1 (Spag1), mRNA. |
| ILMN_1221178 | PDLIM2 | -2.07 | 14 | Mus musculus PDZ and LIM domain 2 (Pdlim2), mRNA. |
| ILMN_2701631 | SPINK2 | -2.08 | 5 | Mus musculus serine peptidase inhibitor, Kazal type 2 (Spink2), mRNA. |
| ILMN_1237572 | AARD | -2.10 | 15 | Mus musculus alanine and arginine rich domain containing protein (Aard), mRNA. |
| ILMN_2693940 | PSMB8 | -2.11 | 17 | Mus musculus proteasome (prosome, macropain) subunit, beta type 8 (large multifunctional peptidase 7) (Psmb8), mRNA. |
| ILMN_2440823 | TNXB | -2.11 | 17 | Mus musculus tenascin XB (Tnxb), mRNA. |
| ILMN_3115472 | AQP5 | -2.12 | 15 | Mus musculus aquaporin 5 (Aqp5), mRNA. |
| ILMN_2741114 | GUSB | -2.12 | 5 | Mus musculus glucuronidase, beta (Gusb), mRNA. |
| ILMN_1228366 | CDKN2C | -2.12 | 4 | Mus musculus cyclin-dependent kinase inhibitor 2C (p18, inhibits CDK4) (Cdkn2c), mRNA. |
| ILMN_2548010 | HOPX | -2.13 | 5 | Mus musculus HOP homeobox (Hopx), mRNA. |
| ILMN_2594521 | NSDHL | -2.13 | X | Mus musculus NAD(P) dependent steroid dehydrogenase-like (Nsdhl), mRNA. |
| ILMN_1254653 | ANGPTL2 | -2.15 | 2 | Mus musculus angiopoietin-like 2 (Angptl2), mRNA. |
| ILMN_2638114 | PTN | -2.18 | 6 | Mus musculus pleiotrophin (Ptn), mRNA. |
| ILMN_2815506 | GAMT | -2.18 | 10 | Mus musculus guanidinoacetate methyltransferase (Gamt), mRNA. |
| ILMN_2589768 | HTR2B | -2.19 | 1 | Mus musculus 5-hydroxytryptamine (serotonin) receptor 2B (Htr2b), mRNA. |
| ILMN_1238603 | PCOLCE2 | -2.23 | 9 | Mus musculus procollagen C-endopeptidase enhancer 2 (Pcolce2), mRNA. |
| ILMN_1227596 | PMVK | -2.23 | 3 | Mus musculus phosphomevalonate kinase (Pmvk), mRNA. |
| ILMN_1241211 | GUCY1A3 | -2.25 | 3 | Mus musculus guanylate cyclase 1, soluble, alpha 3 (Gucy1a3), mRNA. |
| ILMN_2605890 | TK1 | -2.25 | 11 | Mus musculus thymidine kinase 1 (Tk1), mRNA. |
| ILMN_2754222 | RPP25 | -2.26 | 9 | Mus musculus ribonuclease P 25 subunit (human) (Rpp25), mRNA. |
| ILMN_1233455 | OLFML3 | -2.27 | 3 | Mus musculus olfactomedin-like 3 (Olfml3), mRNA. |
| ILMN_2974611 | TAPBPL | -2.29 | 6 | Mus musculus TAP binding protein-like (Tapbpl), mRNA. |
| ILMN_1225730 | FDPS | -2.30 | 3 | Mus musculus farnesyl diphosphate synthetase (Fdps), mRNA. |
| ILMN_2890935 | AVPR1A | -2.30 | 10 | Mus musculus arginine vasopressin receptor 1A (Avpr1a), mRNA. |
| ILMN_1259174 | SCIN | -2.32 | 12 | Mus musculus scinderin (Scin), mRNA. |
| ILMN_2892507 | FUT4 | -2.33 | 9 | Mus musculus fucosyltransferase 4 (Fut4), mRNA. |
| ILMN_2760254 | MRGPRF | -2.35 | 7 | Mus musculus MAS-related GPR, member F (Mrgprf), mRNA. |
| ILMN_2984012 | FDXR | -2.36 | 11 | Mus musculus ferredoxin reductase (Fdxr), nuclear gene encoding mitochondrial protein, mRNA. |
| ILMN_2695143 | CAPN6 | -2.36 | X | Mus musculus calpain 6 (Capn6), mRNA. |
| ILMN_3139168 | EEF1D | -2.42 | 15 | Mus musculus eukaryotic translation elongation factor 1 delta (guanine nucleotide exchange protein) (Eef1d), transcript variant 1, mRNA. |
| ILMN_2915671 | KCNAB1 | -2.42 | 3 | Mus musculus potassium voltage-gated channel, shaker-related subfamily, beta member 1 (Kcnab1), mRNA. |
| ILMN_2589651 | ANPEP | -2.43 | 7 | Mus musculus alanyl (membrane) aminopeptidase (Anpep), mRNA. |
| ILMN_1228752 | ISYNA1 | -2.45 | 8 | Mus musculus myo-inositol 1-phosphate synthase A1 (Isyna1), mRNA. |
| ILMN_1243254 | ADAM12 | -2.52 | 7 | Mus musculus a disintegrin and metallopeptidase domain 12 (meltrin alpha) (Adam12), mRNA. |
| ILMN_1246139 | CLDN11 | -2.54 | 3 | Mus musculus claudin 11 (Cldn11), mRNA. |
| ILMN_1225565 | IGF1 | -2.55 | 10 | Mus musculus insulin-like growth factor 1 (Igf1), transcript variant 1, mRNA. |
| ILMN_1219942 | FDXR | -2.59 | 11 | Mus musculus ferredoxin reductase (Fdxr), nuclear gene encoding mitochondrial protein, mRNA. |
| ILMN_2742279 | DEFB19 | -2.59 | 2 | Mus musculus defensin beta 19 (Defb19), mRNA. |
| ILMN_2699052 | NRN1 | -2.61 | 13 | Mus musculus neuritin 1 (Nrn1), mRNA. |
| ILMN_2769884 | IGF1 | -2.65 | 10 | Mus musculus insulin-like growth factor 1 (Igf1), transcript variant 1, mRNA. |
| ILMN_2614161 | LSS | -2.65 | 10 | Mus musculus lanosterol synthase (Lss), mRNA. |
| ILMN_2851288 | NGFR | -2.68 | 11 | Mus musculus nerve growth factor receptor (TNFR superfamily, member 16) (Ngfr), mRNA. |
| ILMN_2857666 | COL24A1 | -2.69 | 3 | Mus musculus collagen, type XXIV, alpha 1 (Col24a1), mRNA. XM_916101 |
| ILMN_2754551 | DPEP1 | -2.75 | 8 | Mus musculus dipeptidase 1 (renal) (Dpep1), mRNA. |
| ILMN_1233848 | COPG | -2.75 | 6 | Mus musculus coatomer protein complex, subunit gamma (Copg), transcript variant 2, mRNA. |
| ILMN_2602938 | SMPDL3B | -2.78 | 4 | Mus musculus sphingomyelin phosphodiesterase, acid-like 3B (Smpdl3b), mRNA. |
| ILMN_2661422 | RAMP2 | -2.81 | 11 | Mus musculus receptor (calcitonin) activity modifying protein 2 (Ramp2), mRNA. |
| ILMN_3001540 | LUM | -2.87 | 10 | Mus musculus lumican (Lum), mRNA. |
| ILMN_2645275 | MVD | -2.88 | 8 | Mus musculus mevalonate (diphospho) decarboxylase (Mvd), mRNA. |
| ILMN_1260061 | D17H6S56E-5 | -2.90 | 17 | Mus musculus DNA segment, Chr 17, human D6S56E 5 (D17H6S56E-5), mRNA. |
| ILMN_2512663 | WNT4 | -2.92 | 4 | Mus musculus wingless-related MMTV integration site 4 (Wnt4), mRNA. |
| ILMN_2647594 | IGF1 | -2.99 | 10 | Mus musculus insulin-like growth factor 1 (Igf1), transcript variant 2, mRNA. |
| ILMN_2889641 | WNT4 | -3.05 | 4 | Mus musculus wingless-related MMTV integration site 4 (Wnt4), mRNA. |
| ILMN_2994995 | LGI3 | -3.07 | 14 | Mus musculus leucine-rich repeat LGI family, member 3 (Lgi3), mRNA. |
| ILMN_2983624 | CCRL1 | -3.13 | 9 | Mus musculus chemokine (C-C motif) receptor-like 1 (Ccrl1), mRNA. |
| ILMN_1253062 | INSC | -3.38 | 7 | Mus musculus inscuteable homolog (Drosophila) (Insc), mRNA. |
| ILMN_1225073 | CTHRC1 | -3.47 | 15 | Mus musculus collagen triple helix repeat containing 1 (Cthrc1), mRNA. |
| ILMN_2757617 | IL1RL1 | -3.57 | 1 | Mus musculus interleukin 1 receptor-like 1 (Il1rl1), transcript variant 2, mRNA. |
| ILMN_1231275 | COL24A1 | -3.60 | 3 | Mus musculus collagen, type XXIV, alpha 1 (Col24a1), mRNA. XM_916101 |
| ILMN_2731901 | S100A4 | -3.96 | 3 | Mus musculus S100 calcium binding protein A4 (S100a4), mRNA. |
| ILMN_2830611 | ACCN3 | -4.03 | 5 | Mus musculus amiloride-sensitive cation channel 3 (Accn3), mRNA. |
| ILMN_2834379 | TGFBI | -4.41 | 13 | Mus musculus transforming growth factor, beta induced (Tgfbi), mRNA. |
| ILMN_1259967 | PRL2C2 | -5.48 | 13 | Mus musculus prolactin family 2, subfamily c, member 2 (Prl2c2), mRNA. |
| ILMN_2875585 | PRL2C3 | -6.22 | 13 | Mus musculus prolactin family 2, subfamily c, member 3 (Prl2c3), mRNA. |
| ILMN_2996648 | PRL2C4 | -6.56 | 13 | Mus musculus prolactin family 2, subfamily c, member 4 (Prl2c4), mRNA. |
| ILMN_2617996 | PRL2C2 | -6.67 | 13 | Mus musculus prolactin family 2, subfamily c, member 2 (Prl2c2), mRNA. |
