# Supplementary material for: Amniotic fluid mesenchymal stem cells repair mouse corneal cold injury by promoting mRNA N4-acetylcytidine modification and ETV4/JUN/CCND2 signal axis activation
Source: Hum Cell. 2020 Oct 3;34(1):86–98. doi: 10.1007/s13577-020-00442-7 (PMC7788028; doi:10.1007/s13577-020-00442-7)
Supplement: Supplementary file 4 — Supplementary file4 (DOCX 19 kb) [file 13577_2020_442_MOESM4_ESM.docx]

**Supplementary table 4. Gene Ontology (GO) terms**

| **Category** | **GO ID** | **Term** | **Transcriptional domain coverage (%)** | **p-Value** |
| --- | --- | --- | --- | --- |
| GO Biological Process | GO:0051252 | regulation of RNA metabolic process | 9.451 | 0.002 |
| GO Biological Process | GO:0006355 | regulation of transcription, DNA-dependent | 9.341 | 0.002 |
| GO Biological Process | GO:0042127 | regulation of cell proliferation | 3.956 | 0.002 |
| GO Biological Process | GO:0043009 | chordate embryonic development | 3.571 | <0.001 |
| GO Biological Process | GO:0010628 | positive regulation of gene expression | 3.462 | 0.008 |
| GO Biological Process | GO:0045941 | positive regulation of transcription | 3.352 | 0.01 |
| GO Biological Process | GO:0033554 | cellular response to stress | 3.187 | 0.001 |
| GO Biological Process | GO:0009890 | negative regulation of biosynthetic process | 3.132 | 0.008 |
| GO Biological Process | GO:0032504 | multicellular organism reproduction | 2.967 | 0.009 |
| GO Biological Process | GO:0048609 | reproductive process in a multicellular organism | 2.967 | 0.009 |
| GO Biological Process | GO:0030182 | neuron differentiation | 2.967 | 0.006 |
| GO Biological Process | GO:0048598 | embryonic morphogenesis | 2.857 | 0.002 |
| GO Biological Process | GO:0032989 | cellular component morphogenesis | 2.802 | 0.002 |
| GO Biological Process | GO:0000902 | cell morphogenesis | 2.692 | <0.001 |
| GO Biological Process | GO:0003006 | reproductive developmental process | 2.637 | <0.001 |
| GO Biological Process | GO:0007389 | pattern specification process | 2.582 | <0.001 |
| GO Biological Process | GO:0030030 | cell projection organization | 2.527 | 0.003 |
| GO Biological Process | GO:0035295 | tube development | 2.527 | <0.001 |
| GO Biological Process | GO:0048666 | neuron development | 2.308 | 0.005 |
| GO Biological Process | GO:0006974 | response to DNA damage stimulus | 2.308 | 0.004 |
| GO Biological Process | GO:0048729 | tissue morphogenesis | 2.253 | <0.001 |
| GO Biological Process | GO:0001568 | blood vessel development | 2.143 | 0.001 |
| GO Biological Process | GO:0048568 | embryonic organ development | 2.143 | 0.001 |
| GO Molecular Function | GO:0043167 | ion binding | 21.87 | 0.01 |
| GO Molecular Function | GO:0046872 | metal ion binding | 21.37 | 0.012 |
| GO Molecular Function | GO:0000166 | nucleotide binding | 14.34 | <0.001 |
| GO Molecular Function | GO:0008270 | zinc ion binding | 12.36 | 0.007 |
| GO Molecular Function | GO:0017076 | purine nucleotide binding | 12.2 | <0.001 |
| GO Molecular Function | GO:0032555 | purine ribonucleotide binding | 11.81 | <0.001 |
| GO Molecular Function | GO:0032553 | ribonucleotide binding | 11.81 | <0.001 |
| GO Molecular Function | GO:0003677 | DNA binding | 11.1 | 0.001 |
| GO Molecular Function | GO:0001882 | nucleoside binding | 9.67 | 0.002 |
| GO Molecular Function | GO:0001883 | purine nucleoside binding | 9.56 | 0.003 |
| GO Molecular Function | GO:0030554 | adenyl nucleotide binding | 9.56 | 0.002 |
| GO Molecular Function | GO:0032559 | adenyl ribonucleotide binding | 9.18 | 0.002 |
| GO Molecular Function | GO:0005524 | ATP binding | 9.18 | 0.001 |
| GO Molecular Function | GO:0030528 | transcription regulator activity | 7.8 | 0.001 |
| GO Molecular Function | GO:0003700 | transcription factor activity | 5.55 | <0.001 |
| GO Molecular Function | GO:0043565 | sequence-specific DNA binding | 3.96 | 0.003 |
| GO Molecular Function | GO:0019001 | guanyl nucleotide binding | 2.97 | <0.001 |
| GO Molecular Function | GO:0032561 | guanyl ribonucleotide binding | 2.97 | <0.001 |
| GO Molecular Function | GO:0005525 | GTP binding | 2.91 | <0.001 |
| GO Molecular Function | GO:0060589 | nucleoside-triphosphatase regulator activity | 2.69 | 0.008 |
| GO Molecular Function | GO:0030695 | GTPase regulator activity | 2.64 | 0.009 |
| GO Molecular Function | GO:0048037 | cofactor binding | 2.03 | 0.001 |
| GO Molecular Function | GO:0005083 | small GTPase regulator activity | 1.81 | 0.007 |
| GO Molecular Function | GO:0008083 | growth factor activity | 1.59 | <0.001 |
| GO Cell Component | GO:0051252 | regulation of RNA metabolic process | 9.451 | 0.002 |
| GO Cell Component | GO:0006355 | regulation of transcription, DNA-dependent | 9.341 | 0.002 |
| GO Cell Component | GO:0042127 | regulation of cell proliferation | 3.956 | 0.002 |
| GO Cell Component | GO:0009792 | embryonic development ending in birth or egg hatching | 3.626 | <0.001 |
| GO Cell Component | GO:0043009 | chordate embryonic development | 3.571 | <0.001 |
| GO Cell Component | GO:0010628 | positive regulation of gene expression | 3.462 | 0.008 |
| GO Cell Component | GO:0045941 | positive regulation of transcription | 3.352 | 0.01 |
| GO Cell Component | GO:0033554 | cellular response to stress | 3.187 | 0.001 |
| GO Cell Component | GO:0009890 | negative regulation of biosynthetic process | 3.132 | 0.008 |
| GO Cell Component | GO:0031327 | negative regulation of cellular biosynthetic process | 3.132 | 0.007 |
| GO Cell Component | GO:0032504 | multicellular organism reproduction | 2.967 | 0.009 |
| GO Cell Component | GO:0048609 | reproductive process in a multicellular organism | 2.967 | 0.009 |
| GO Cell Component | GO:0030182 | neuron differentiation | 2.967 | 0.006 |
| GO Cell Component | GO:0048598 | embryonic morphogenesis | 2.857 | 0.002 |
| GO Cell Component | GO:0032989 | cellular component morphogenesis | 2.802 | 0.002 |
| GO Cell Component | GO:0000902 | cell morphogenesis | 2.692 | <0.001 |
| GO Cell Component | GO:0003006 | reproductive developmental process | 2.637 | <0.001 |
| GO Cell Component | GO:0007389 | pattern specification process | 2.582 | <0.001 |
| GO Cell Component | GO:0030030 | cell projection organization | 2.527 | 0.003 |
| GO Cell Component | GO:0035295 | tube development | 2.527 | <0.001 |
| GO Cell Component | GO:0048666 | neuron development | 2.308 | 0.005 |
