# Supplementary figures and images for: Amniotic fluid mesenchymal stem cells repair mouse corneal cold injury by promoting mRNA N4-acetylcytidine modification and ETV4/JUN/CCND2 signal axis activation
Source: Hum Cell. 2020 Oct 3;34(1):86–98. doi: 10.1007/s13577-020-00442-7 (PMC7788028; doi:10.1007/s13577-020-00442-7)

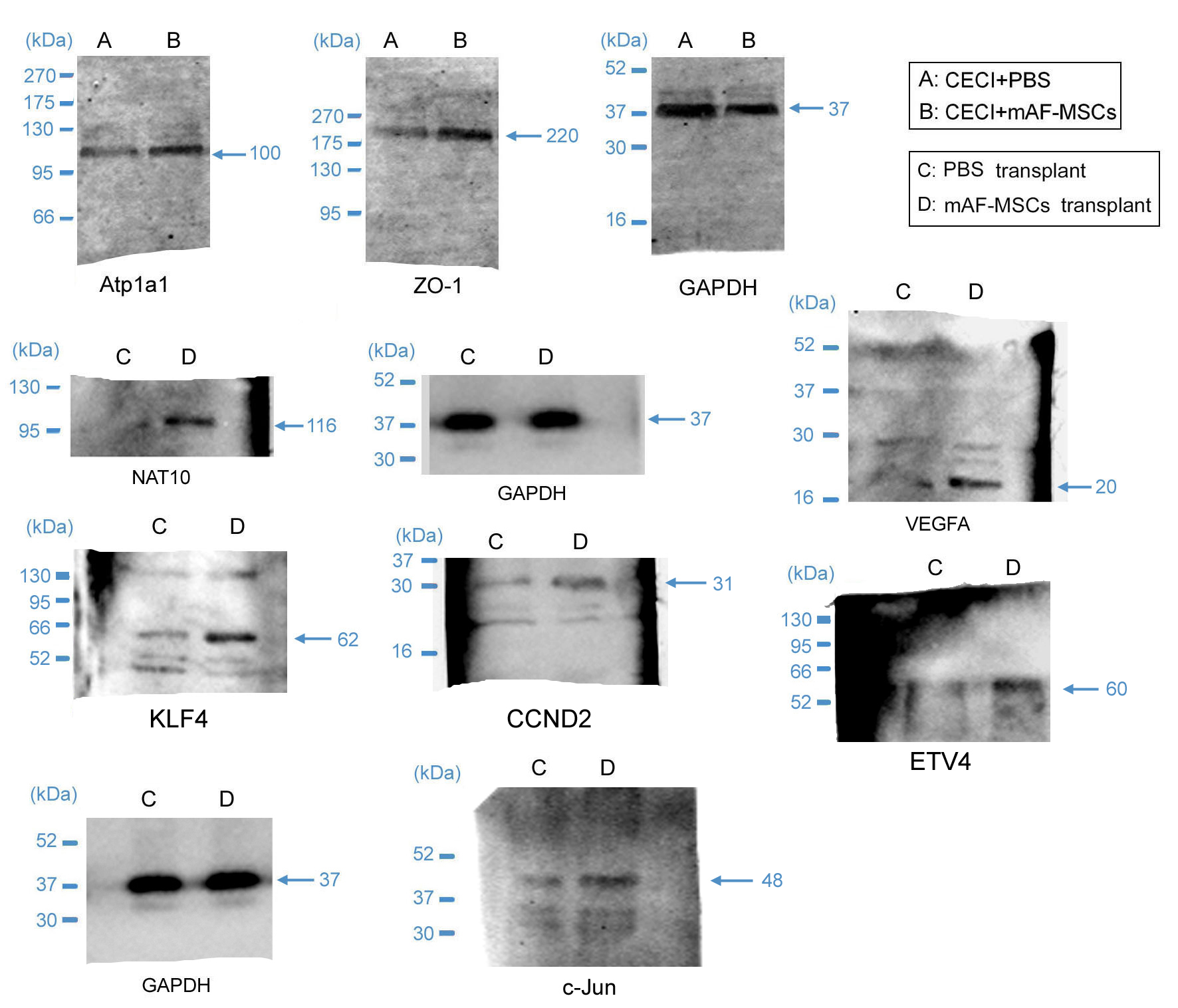

Supplement: Supplementary file 5 — Supplementary file5 (JPG 574 kb) [file 13577_2020_442_MOESM5_ESM.jpg]
